# Assessing local chlamydia screening performance by combining survey and administrative data to account for differences in local population characteristics

**DOI:** 10.1038/s41598-019-43521-y

**Published:** 2019-05-08

**Authors:** Nathan Green, Ellie Sherrard-Smith, Clare Tanton, Pam Sonnenberg, Catherine H. Mercer, Peter J. White

**Affiliations:** 1 MRC Centre Global Infectious Disease Analysis, Department of Infectious Disease Epidemiology, School of Public Health, Faculty of Medicine, Imperial College London, Norfolk Place, London, W2 1PG UK; 2 NIHR Health Protection Research Unit in Modelling Methodology, Department of Infectious Disease Epidemiology, School of Public Health, Faculty of Medicine, Imperial College London, Norfolk Place, London, W2 1PG UK; 3 Centre for Population Research in Sexual Health & HIV, Institute for Global Health, University College London, Mortimer Market Centre, off Capper Street, London, WC1E 6JB UK; 4 Modelling and Economics Unit, National Infection Service, Public Health England, London, NW9 5EQ UK

**Keywords:** Bacterial infection, Risk factors

## Abstract

Reducing health inequalities requires improved understanding of the causes of variation. Local-level variation reflects differences in local population characteristics and health system performance. Identifying low- and high-performing localities allows investigation into these differences. We used Multilevel Regression with Post-stratification (MRP) to synthesise data from multiple sources, using chlamydia testing as our example. We used national probability survey data to identify individual-level characteristics associated with chlamydia testing and combined this with local-level census data to calculate expected levels of testing in each local authority (LA) in England, allowing us to identify LAs where observed chlamydia testing rates were lower or higher than expected, given population characteristics. Taking account of multiple covariates, including age, sex, ethnicity, student and cohabiting status, 5.4% and 3.5% of LAs had testing rates higher than expected for 95% and 99% posterior credible intervals, respectively; 60.9% and 50.8% had rates lower than expected. Residual differences between observed and MRP expected values were smallest for LAs with large proportions of non-white ethnic populations. London boroughs that were markedly different from expected MRP values (≥90% posterior exceedance probability) had actively targeted risk groups. This type of synthesis allows more refined inferences to be made at small-area levels than previously feasible.

## Introduction

Health inequalities are associated with social inequalities, which are strongly linked to geographic location^1^. The UK-government commissioned Marmot Review^1^ recommended that health interventions must be universal but with a scale and intensity proportionate to the level of disadvantage in an area. Achieving this requires an understanding of health inequalities at the local level but most observational data are not (directly) relevant for addressing public health questions at both a national level and for sub-populations. Taking the simple approach to assessing performance of comparing local areas with the average of all local areas to see which ones are above or below average, and by how much, effectively assumes that the composition of each local area’s population is the same, when in fact the populations can be very different. One solution is to join individual-level data with area-level data to create a multilevel (or hierarchical) dataset^2^. However, this is restricted by the relatively small sample sizes and coverage of individual-level data. To address this, a popular approach in geography and machine learning is synthetic reconstruction or reweighting to generate micro data, that is, spatially-detailed individual-level data^3^. However, these are not principled statistical approaches and do not provide additional understanding of underlying behaviours, relationships and associations. In the social sciences, Park *et al*.^4^ introduced a framework which fits a multilevel logistic regression model to individual-level data conditional on post-stratification proportions from the area-level data, often called Multilevel Regression with Post-stratification (MRP). In this paper, we describe how we adapted this method^4^ in order to synthesise data from multiple sources and then compared the model’s results with national recorded surveillance data, with the intention of improving the evidence-base with which to inform local planning and assessment of health inequalities. We use chlamydia testing as our example because the need to use “good local data […] to develop plans to improve local sexual health outcomes and reduce health inequalities” is explicitly recognised in England^5^. Surveillance data show that annual chlamydia testing in 15–24 year olds varies widely by locality (local authorities, LAs) in England, ranging from 10% (Waveney) to 66% (Kensington and Chelsea) (England average 23%)^6^. There is also marked variation in the prevalence and incidence of chlamydia amongst LAs^7^. Use of the MRP approach enables us to understand how much of this variation may be appropriate if explained by sociodemographic and behavioural differences in the LA populations in contrast to inequalities in intervention delivery. In a similar way to using exceedance of ‘control limits’ on a funnel plot to identify outlier institutional performance^8^, LAs with marked deviation of rates of recorded testing from expected rates obtained by MRP estimates could be investigated to learn reasons for their performance being lower or higher than expected, such as use of innovative approaches to providing access to testing^9^ and in partner management^10^. This approach, to our knowledge, has never been used in an infectious disease context in England.

## Methods

We focused on individual-level social and demographic factors previously identified to be associated with chlamydia testing in the third British National Survey of Sexual Attitudes and Lifestyles (Natsal-3), a nationally-representative probability sample survey of 15,162 people aged 16–74 conducted in 2010–2012^11,12^. Data were available from Natsal-3 for these factors for England at four geographic levels: individual, LA (*n* = 326), county (*n* = 83) and regional (*n* = 8: East of England, East Midlands, London, North East, North West, South East, South West and West Midlands). A multilevel logistic regression model was then fitted to the Natsal-3 individual-level data conditional on post-stratification proportions from the area-level census and administrative data^4^. Finally, the model was used to estimate the level of testing in each LA given its population characteristics. These expected levels of testing were then compared to recorded testing surveillance data in each LA.

### Data collation

#### Natsal-3

The Natsal-3 data are weighted to be representative of the English population with respect to sex, age, and regional distribution^13,14^ and were used to identify and quantify individual-level characteristics and behaviours associated with the probability of an individual reporting having been tested for chlamydia in the last year.

#### Demographic and risk factor data

All aggregated LA-level or age-sex grouped data were openly-available from the Office for National Statistics (ONS), either from the 2011 census^15^ or routinely-collected administrative datasets^16–20^.

#### Surveillance data

Comprehensive chlamydia testing surveillance data for 2011 (to align the surveillance data with the data collection period for Natsal-3) were obtained from the National Chlamydia Screening Programme (NCSP, which tests 15–24-year-olds), Genitourinary Medicine Clinic Activity Dataset (GUMCAD, which records testing of all ages) and non-NCSP and non-GUMCAD dataset (NNNG, which records testing up to 24 years old)^21^. As per recommendations from Woodhall *et al*.^22^ we scaled the LA testing proportions from the surveillance data by 0.95 to account for errors in the data, including double-counting across datasets and repeat tests (see Supplementary Material).

### Statistical analysis

#### Individual-level logistic regression model using Natsal-3

Natsal-3 response for each individual *i* is denoted by *y*_*i*_, where *y*_*i*_ = 1 represents the participant reporting testing for chlamydia in the last year and 0 otherwise. Individual-level covariates from Natsal-3 were sex (male/female), ethnicity (White, Black/Black British, Asian/Asian British, Chinese, Mixed, Other), current full-time student status (yes/no), whether an individual lives alone (household size one) and age (years). Covariates were chosen because of known STI risk factors^23^, availability in individual and area-level data sets, use in survey design^14^, posterior predictions and model selection statistics Akaike Information Criterion (AIC) and Bayesian Information Criterion (BIC)^24^ were produced. Note that this is only a guide since standard model-selection approaches are complicated for multilevel models^25^. Also, covariates with apparently small effect sizes may have a larger influence in the post-stratification step. The LA-level covariates were the upper quintile Index of Multiple Deprivation (IMD) i.e. identifying the most deprived areas relative to all others, ≤18 years old conception rate per 1000 women and the ONS urban-rural area classification (Major Urban, Large Urban, Other Urban, Significant Rural, Rural-50, Rural-80, where Rural-50 and -80 are those areas which have ≥50% and ≥80% of their population living in a rural area, respectively). These multilevel data were used in the model:

1 $$Pr({y}_{i}=\mathrm{1)}={{\rm{logit}}}^{-1}({\beta }^{0}+{\alpha }_{j[i]}^{age}+{\alpha }_{i}^{male}mal{e}_{i}+{\alpha }_{k[i]}^{ethnicity}+{\alpha }_{i}^{student}studen{t}_{i}+{\alpha }_{i}^{livealone}livealon{e}_{i}+{\alpha }_{m[i]}^{la}+{\varphi }_{m[i]}^{la}),$$

2 $${\alpha }_{m}^{la}\sim N({\alpha }_{m}^{IMD}IM{D}_{m}+{\alpha }_{j[m]}^{ONSclass}+{\alpha }_{l[m]}^{county}+{\alpha }_{p[m]}^{conception},{\sigma }_{la}^{2}),\,m=1,\ldots ,326\,{\rm{local}}\,{\rm{authorities}},$$

3 $${\alpha }_{l}^{county}\sim N({\alpha }_{q[l]}^{gors},{\sigma }_{county}^{2}),\,l=1,\ldots ,83\,{\rm{counties}},$$

where, *e*^*α*^ represents the multiplicative effects on the odds ratios (ORs) for the respective covariates that describe the probability of testing. In the Bayesian model, independent normal distributions centred at 0 with standard deviations σ estimated from the data are assigned to the varying covariates, allowing a multilevel structure. The ${\varphi }_{m}^{la}$ is the additional LA-structured random effect whose distribution conditions on the value of ${\varphi }_{m}^{la}$ in the neighbouring LAs. We used a conditionally autoregressive (CAR) distribution (details in the Supplementary Material). The remaining, fixed effect coefficients were not modelled with a multilevel structure. The values of female, non-student, not most deprived quintile, and cohabiting were set as fixed effect coefficient baseline. The Natsal-3 complex survey design was accounted for by including the covariates that have an effect on sampling or nonresponse in the regression model (age, sex, region and household size one)^26^.

#### Model Fitting

Bayesian models with uninformative priors were fitted using a Gibbs Markov chain Monte Carlo sampling algorithm implemented using the software *R* version 3.4.4^27^ and WinBUGS^28^. Three Markov chains were initialised to assess convergence; the first 2000 iterations were discarded as burn-in. The posterior distributions were formed from 100,000 iterations with a thinning rate of 250 to estimate coefficients and generate 50% and 95% Bayesian credible intervals (CrI) for the model fits.

#### Post-stratification categories

Ideally, to perform the post-stratification a discrete joint distribution is required over all combinations of covariate values i.e. categories (for the individual-level variables, age (9 levels), sex (2 levels), ethnicity (6 levels), student status (2 levels) and living alone (2 levels) within a given LA, is a total of 432 combinations). However, allowing progressively higher dimensions reduces the subgroup sample sizes. Further, these combined data were not available. Instead, a simple LA adjustment (relative to the national average) was used to weight the data to account for LA, age and sex. As an example, if an LA had twice as many students as the national average, then the probability of being a student given age and sex was adjusted by a factor of two. It was then assumed that the variables were conditionally independent of one another given LA, age and sex of an individual. This allows estimates for the overall category probabilities to be obtained from the product of the conditional probabilities (see Supplementary Material).

#### LA-level estimates by post-stratification

Using the multilevel model described above, mean estimates of testing for chlamydia were obtained for all post-stratification categories. The LA-level estimates were acquired by summing the predicted individual probabilities over the categories and weighting by the categories corresponding to subpopulation sizes within each LA. For each category *j*, the estimated population average of the probability of testing for chlamydia in the last year in LA *S* was then:

4 $${p}_{la}=\frac{\sum _{j\in S}\,{N}_{j}{p}_{j}}{\sum _{j\in S}\,{N}_{j}}$$

with each summation over all possible categories in an LA.

## Results

### Bayesian regression individual-level model

**Figure 1:**
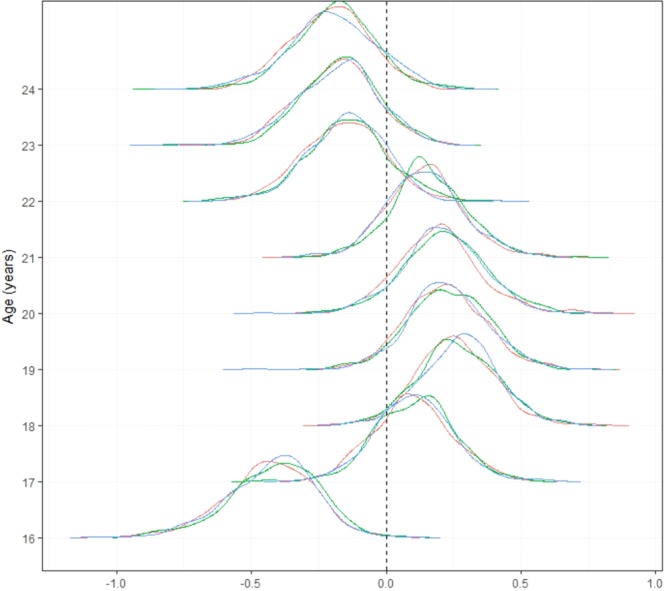
Full posterior distributions of the log odds ratio of probability of chlamydia testing in previous year by age estimated using a Bayesian multilevel regression model. Estimates from multiple Markov chain Monte Carlo (MCMC) chains are shown for each age (green, blue and red lines).

**Figure 2:**
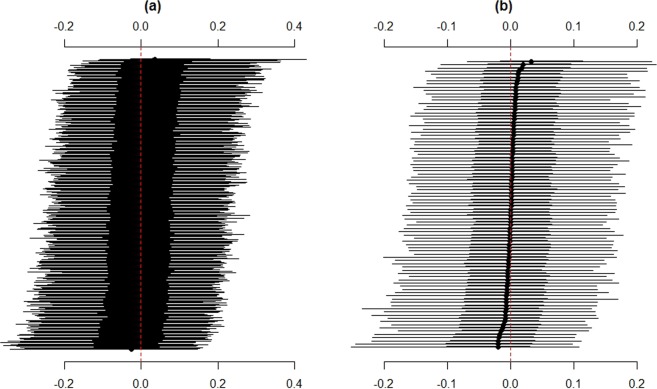
Posterior distributions of the log odds ratio for **(a)** local authorities (LAs) and **(b)** counties, estimated using a Bayesian multilevel regression model. Points indicate posterior modes, bold lines (▬) are the 50% credible intervals and narrow lines (─) are the 95% credible intervals. LAs are ordered from most to least amounts of chlamydia testing.

**Figure 3:**
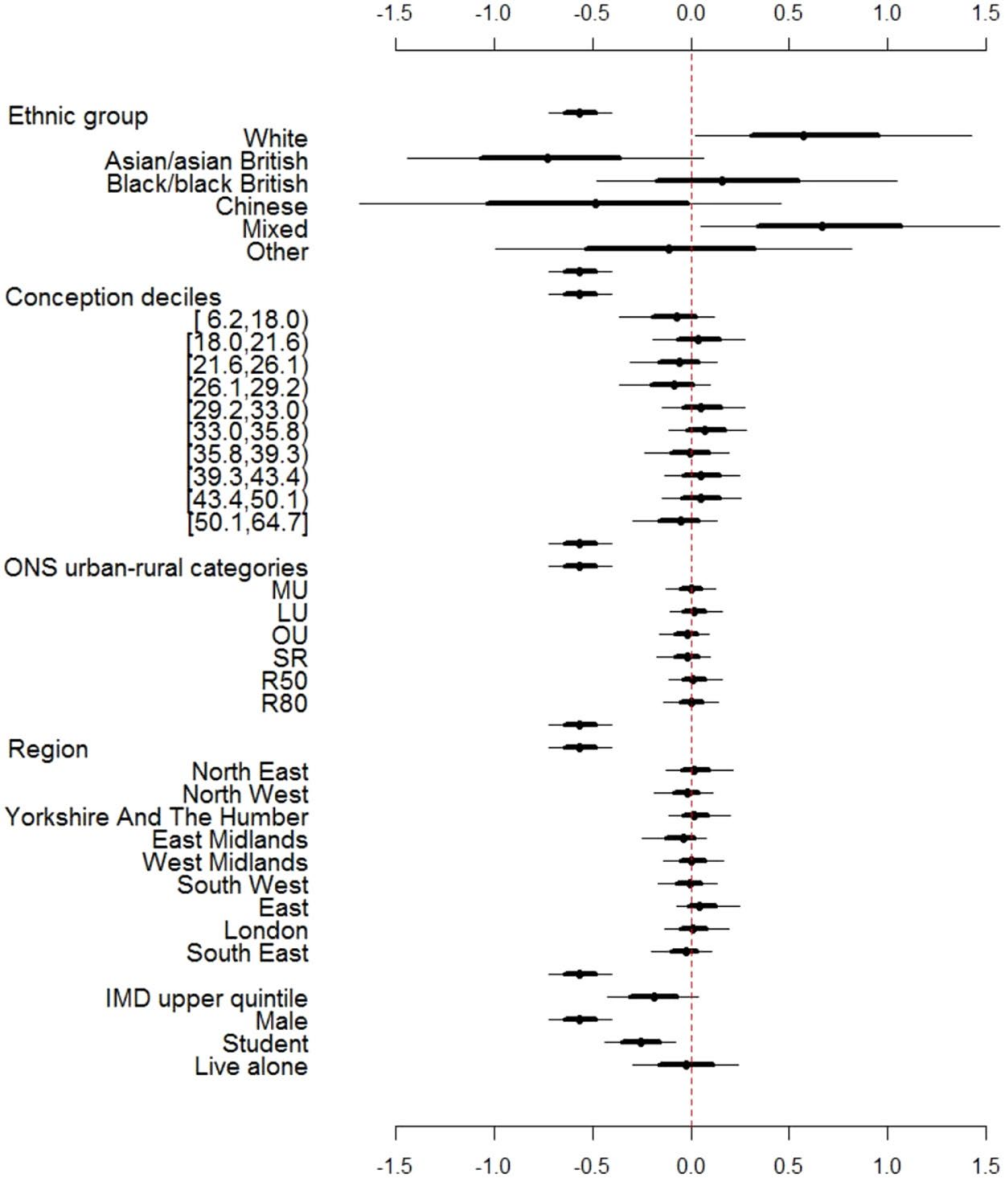
Posterior distributions of the log odds ratio of model parameters, estimated using a Bayesian multilevel regression model. Points indicate posterior modes, bold lines (▬) are the 50% credible intervals and narrow lines (─) are the 95% credible intervals.

The model indicated that the probability of chlamydia testing differed between ethnic groups, with Black, White and Mixed ethnic groups testing more than Asian, Chinese or people of undisclosed ethnicity; however, there were large uncertainties (Fig. 1). For the fixed effects, females were more likely to test than males (estimated OR: 1.73, 95% CI [0.07, 0.68]; corresponding with^29^). Students were less likely to test than non-students (estimated OR: 0.82, 95% CI [0.68, 0.99]). Those in the upper quintile of IMD were slightly less likely to test (OR 0.84, 95% CI [0.66, 1.07] vs. those not in the upper quintile) but data were consistent with there being no association. Living alone had a relatively small association with chlamydia testing (OR: 0.87, 95% CI [0.66, 1.16]). Figure 2 shows posterior distributions for age which show it to be a good predictor of testing behaviour; whilst 16-year olds did not test as much as older participants (which reflects the fact that half of participants aged 16 had not had sex and would not have been at risk of infection), peak testing was estimated for 17–19-year olds and chlamydia testing decreased with older ages. Supplementary Material describes a two-step regression model to account for this pattern. Individuals in LAs with lower conception rates for those ≤18 years old were generally less likely to test for chlamydia. Figure 3 shows the (ordered) LA and county level parameter posterior estimates. Many of the LAs had similar estimates although the CrI were relatively wide. Model checking plots and statistics are given in the Supplementary Material.

### Comparison to recorded testing surveillance data

**Figure 4:**
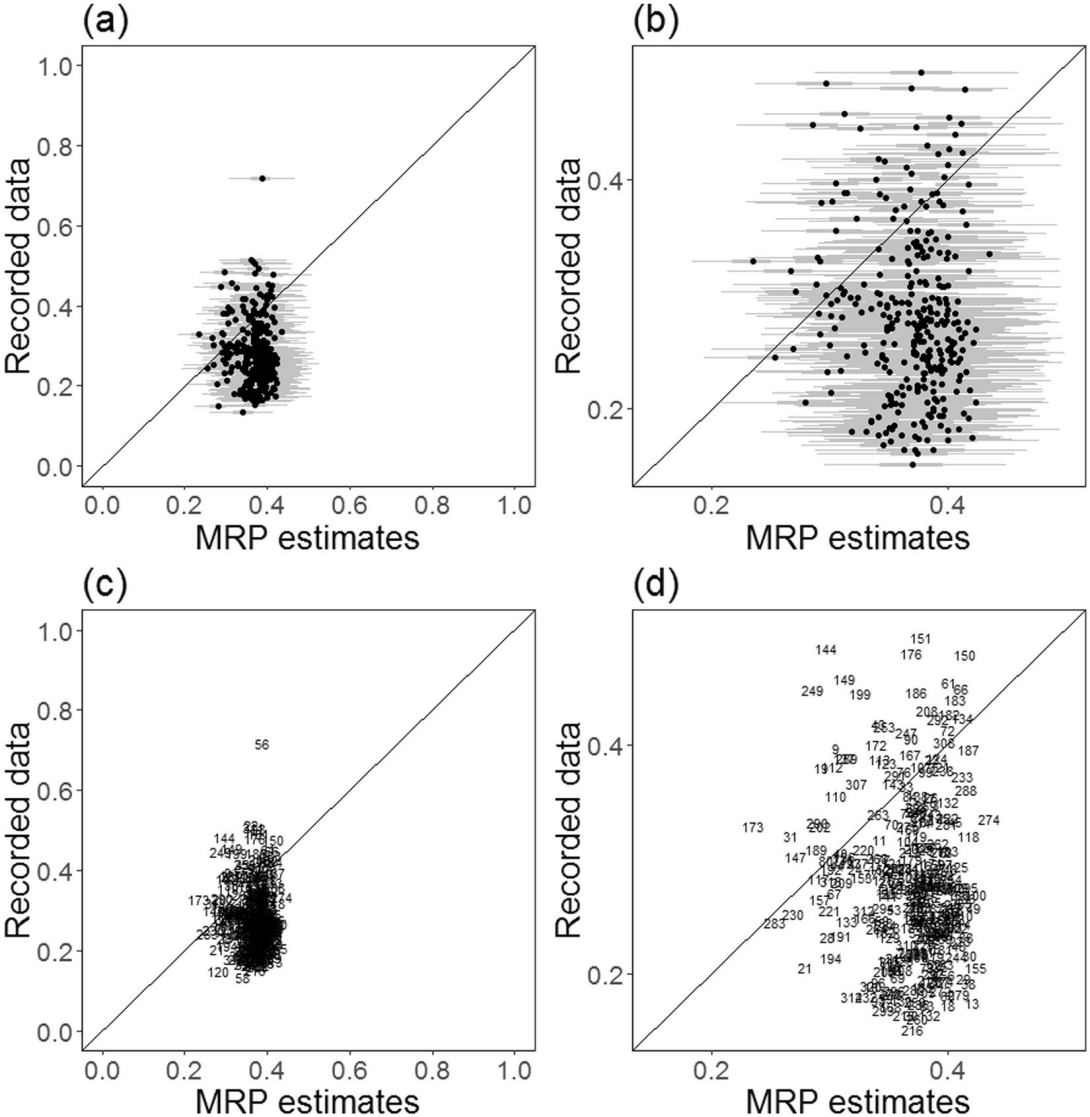
Posterior predictions of chlamydia testing coverage in the previous year using MRP estimates with propagated uncertainty against recorded testing in NCSP 2011 surveillance data, showing **(a)** 50% (thick grey line ()) and 95% (thin grey line ()) credible intervals for each LA propagating forward the uncertainty associated with each parameter estimate, **(b)** zoomed-in Figure a, **(c)** numbered LA points, and **(d)** zoomed-in Figure c. Diagonal lines indicate equality of MRP estimates and recorded testing.

**Figure 5:**
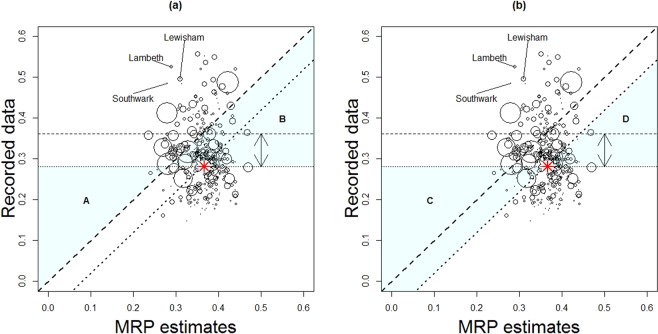
Bubble plot of LA-level MRP point estimates using Bayesian maximum a-posteriori (MAP) estimates of chlamydia testing against 2011 recorded testing rates. Horizontal lines show the weighted mean Natsal-3 coverage (dashed ()) and the mean recorded testing coverage (dotted ()). Diagonal lines show where the estimates produced using Natsal-3 equal the recorded testing (dashed) and where the MRP estimates equal the recorded testing values and adjusting the estimated mean to equal the recorded national average (dotted). The size of this adjustment is indicated by the vertical arrowed line. The England-wide MRP point estimate vs recorded testing is indicated by the red asterisk (). Plot regions are highlighted where an LA performance categorisation depends on whether the recorded testing mean or estimated testing is used () (see text for details). Regions are shown for **(a)** MRP estimates against recorded data; **(b)** recorded data mean shifted MRP estimates against recorded data. The bubble sizes are proportional to the Natsal-3 sample size.

Figure 4 shows the expected level of testing in each LA given the specific characteristics of each locality (estimated from the MRP model) compared to the level of testing recorded in the surveillance data. In England, there were no MRP estimates distant from the others; all 95% CrIs overlapped with at least one other. For many LAs the MRP estimates were higher than recorded testing. This is in-part due to the MRP estimates being distributed about the posterior mean. Furthermore, the variability of MRP estimates was smaller than for recorded testing data. This may be due to the model not distinguishing between different LAs for the given covariates. The model did not appear to estimate higher testing rates than the posterior mean and did distinguish several LAs as being smaller than the mean. In Fig. 5, we see that the England mean averages for chlamydia testing from Natsal-3, 35% (horizontal dashed line) and recorded testing, 26% (horizontal dotted line), suggest that there were discrepancies in the two data sources, even accounting for uncertainties about these estimates. The Natsal-3 average was weighted to account for sample design but may have recall and other biases. The proportion of LAs with rates of recorded testing above the mean rate of recorded testing was 43%, whilst the proportion of LAs with rates of recorded testing above the mean Natsal-based estimated rate of testing was 10%. LAs above the dashed diagonal line had higher rates of testing than expected, given their population demographics, whilst those below these lines had less testing than expected. The proportion of LAs with recorded testing above estimated was 26%. The dotted diagonal line is shifted to have the same mean as the recorded testing rather than the Natsal-3 regression intercept value, *β*^ 0^. Assessment of LA performance could be made using any of these classification lines. The annotation letters indicate plotting regions where an LA classification may change depending on which threshold is being used (see Table 1).

A. Below threshold when using the recorded testing mean but above threshold when using the modelled LA population composition (see Fig. 5a).
B. Above threshold when using the recorded testing mean but below threshold when using the modelled LA population composition (see Fig. 5a).
C. Below threshold when using the recorded testing mean but above threshold when using the modelled LA population composition shifted to have the same mean as the recorded testing (see Fig. 5b).
D. Above threshold when using the recorded testing mean but below threshold when using the LA population composition shifted to have the same mean as the recorded testing (see Fig. 5b).

**Table 1:** Proportion of local authorities (LA) in plotting regions defined by the MRP, adjusted MRP and mean recorded testing thresholds, where ${p}_{la}^{rec}$ are the estimated probability of testing for chlamydia in the previous year for an LA using NCSP 2011, ${\bar{p}}^{rec}$ is the mean of ${p}_{la}^{rec}$, ${p}_{la}^{mrp}$ are the equivalent probabilities calculated using MRP and Natsal-3 data, and ${p}_{la}^{mrp\ast }$ are the MRP estimates adjusted so that ${\bar{p}}^{rec}={\bar{p}}^{mrp\ast }$.

	${{\boldsymbol{p}}}_{{\boldsymbol{la}}}^{{\boldsymbol{rec}}} > {{\boldsymbol{p}}}_{{\boldsymbol{la}}}^{{\boldsymbol{mrp}}}$	${{\boldsymbol{p}}}_{{\boldsymbol{la}}}^{{\boldsymbol{rec}}} < {{\boldsymbol{p}}}_{{\boldsymbol{la}}}^{{\boldsymbol{mrp}}}$	${{\boldsymbol{p}}}_{{\boldsymbol{la}}}^{{\boldsymbol{rec}}} > {{\boldsymbol{p}}}_{{\boldsymbol{la}}}^{{\boldsymbol{mrp}}\ast }$	${{\boldsymbol{p}}}_{{\boldsymbol{la}}}^{{\boldsymbol{rec}}} < {{\boldsymbol{p}}}_{{\boldsymbol{la}}}^{{\boldsymbol{mrp}}\ast }$
${p}_{la}^{rec} > {\bar{p}}^{rec}$	24%	19%	34%	8%
${p}_{la}^{rec} < {\bar{p}}^{rec}$	2%	56%	4%	54%

**Figure 6:**
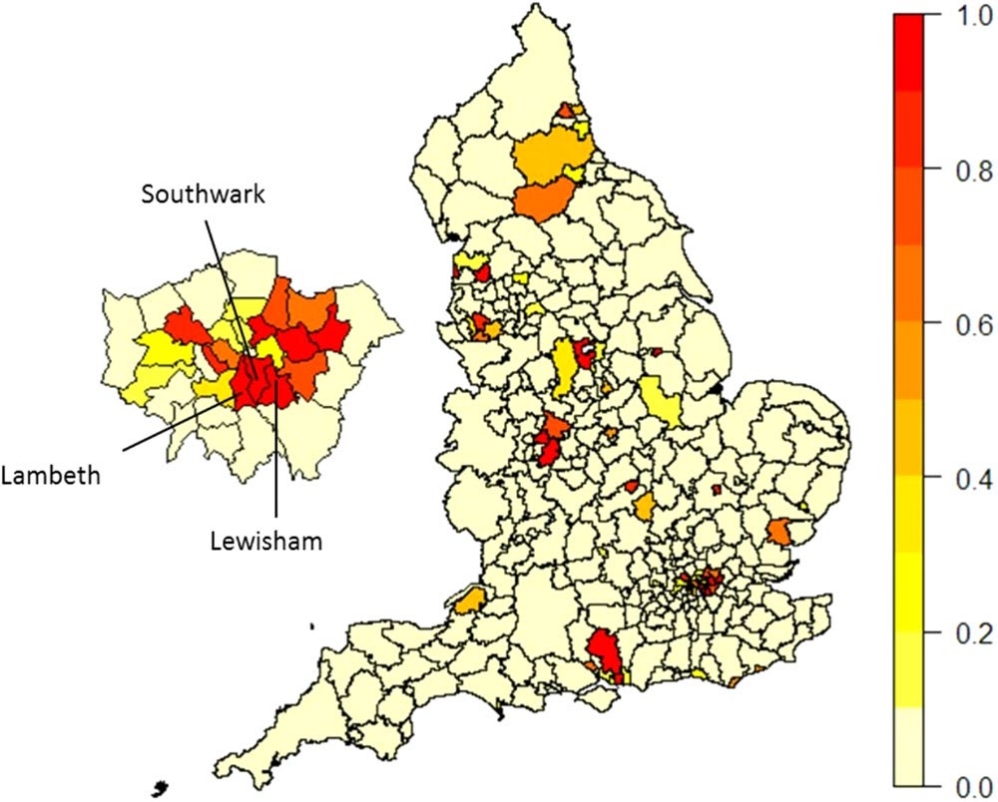
A choropleth map of England by LA of posterior probability of testing for chlamydia in the previous year in NCSP 2011 exceeding Multilevel Regression with Post-stratification model estimates (MRP) (The map was generated in *R* version 3.4.4 (https://www.r-project.org/) using the rgdal and ggplot2 packages).

Figure 6 shows a choropleth map of LAs in England by posterior probability of recorded testing for chlamydia in the previous year exceeding MRP estimates. The red areas are those LAs that had a high likelihood of exceeding expected testing rates given their population’s characteristics. Several of the more-deprived London boroughs had high probability, as well as LAs near other large cities in England, including Birmingham and Manchester. Related plots for specific threshold probabilities are given in the Supplementary Material.

### Specific observations

Recorded testing for the London LAs of Lambeth, Southwark and Lewisham were markedly different from the MRP expected values, such that the chance of the posterior probability of the surveillance values being smaller than the MRP estimates were small (<0.1) (Fig. 6).

## Discussion

Reducing health inequalities and improving the performance of public health systems requires identifying examples of poor performance which require special attention - as is increasingly done with surgical mortality statistics, for example - and identifying examples of high performance, from which lessons can be learned to be applied elsewhere. In turn, this requires comparing observed performance with expected performance. In the case of chlamydia screening rates, we have shown a large variation amongst local authorities (LAs) in England, but there was also large variation in the demographic composition of LA populations and therefore crude comparison of those that are above or below average, or even placing in to quintiles, does not indicate which LAs are performing better or worse than expected, given their populations.

To address this challenge, we used a Multilevel Regression with Post-stratification (MRP) model in order to maximise the utility of data collected by detailed, nationally-representative surveys and national census. To our knowledge, this approach has had limited application to public health research to-date^30,31^ and has never been used in an infectious disease context in England. Previously, comparison of data from Natsal-2 (1999–2001) and NCSP (in 2008) found that NCSP tested a greater proportion of individuals with STI risk factors^32^. The MRP approach used here aimed to adjust for such an imbalanced sample. The numbers of non-White British participants in Natsal-3 was relatively small and may limit the statistical power for subgroup analysis. Uncertainty was explicitly quantified in the posterior distributions but the estimates for these ethnic groups was unavoidably less informative. Another limitation of the data is that neither Natsal-3 nor surveillance data recorded the frequency of repeat testing by individuals. However, analysis of national-level data from the previous year (i.e. 2010)^22^ reported that of people who tested for chlamydia, 89.8% tested once in the year, and 89.2% of tests performed were on people who tested once, so data on retesting would likely have had only a minor effect on our results. A key insight from our study is how much variation amongst LAs was accounted for by variation in measured local population characteristics. However, for binary data, such as STI testing, further research is required to explain variances between levels in multilevel models, e.g. LA and county level^33^.

The MRP method is widely-applicable across public health and can enable researchers and decision-makers to make better use of publicly-funded data to facilitate insights to improve services. With economic austerity and an increasing focus on secondary data analyses, the MRP method can help draw inferences about population behaviours where data are sparse, or a specific study was not performed. Adopting the MRP approach enabled us to identify a subset of LAs where testing rates were different from expected given the characteristics of the population. This is a better way to assess LA performance than crude analysis of testing rates e.g. by ranking areas relative to a pooled national average^34^. Once LAs with particularly high (or low) performance relative to expectations have been identified the underlying reasons can be investigated. These are likely to include local operational decisions, which can be examined to help LAs learn from each other. In conclusion, we have demonstrated use of the MRP approach to chlamydia screening coverage in England. This novel approach enables identification of LAs where chlamydia testing coverage is lower or higher than expected given the characteristics of their populations. We identified that the London boroughs of Lambeth, Southwark and Lewisham out-performed all other London boroughs (as well as most other LAs across England) in chlamydia testing, which suggests that their coordinated sexual health strategy^35,36^ has been successful. With limited evidence of the overall effectiveness of chlamydia screening in England to-date^37^, we recommend that NCSP investigate the particular approaches to promoting and providing screening used by these high-performing LAs to determine what lessons can be learned to improve the performance of other LAs to maximise the benefits of the programme nationally. Improved data, including recording in a future Natsal study and surveillance data why the patient was tested, whether the patient had symptoms (and their duration if applicable), information on the patient’s sexual risk behaviour^7,37^ and the frequency of retesting, with surveillance data broken down by age, sex, and geographic location so that it could be incorporated into analysis of the type presented here, would enable further insights to improve NCSP.

## Supplementary information

Supplementary material

## Data Availability

The datasets analysed during the current study are all publicly available in the Office for National Statistics, Public Health England or UK Data Service repositories^12,15–21^.
