# Supplementary material for: Assessing local chlamydia screening performance by combining survey and administrative data to account for differences in local population characteristics
Source: Sci Rep. 2019 May 8;9:7070. doi: 10.1038/s41598-019-43521-y (PMC6506589; doi:10.1038/s41598-019-43521-y)
Supplement: Supplementary file 1 — Supplementary material [file 41598_2019_43521_MOESM1_ESM.pdf]

Title:

Assessing local chlamydia screening performance by combining survey and administrative data to account for differences in local population characteristics

Nathan Green[1,2*], Ellie Sherrard-Smith[1], Clare Tanton[3], Pam Sonnenberg[3], Catherine H Mercer[3], Peter J White[1,2,4]

***Corresponding author:** Nathan Green nathan.green@imperial.ac.uk

Addresses:

1. MRC Centre Global Infectious Disease Analysis, Department of Infectious Disease Epidemiology, School of Public Health, Faculty of Medicine, Imperial College London, Norfolk Place, London W2 1PG, UK

2. NIHR Health Protection Research Unit in Modelling Methodology, Department of Infectious Disease Epidemiology, School of Public Health, Faculty of Medicine, Imperial College London, Norfolk Place, London W2 1PG, UK

3. Centre for Sexual Health & HIV Research, Research Department of Infection and Population Health, University College London, Mortimer Market Centre, off Capper Street, London WC1E 6JB, UK

4. Modelling and Economics Unit, National Infection Service, Public Health England, London NW9 5EQ, UK

SUPPLEMENTARY MATERIAL

Supporting data and additional results are given in the following sections.

Region	Local authority (LA)	LA number in region	LA number
East Midlands	West Lindsey	1	302
East Midlands	Wellingborough	2	296
East Midlands	South Northamptonshire	3	241
East Midlands	South Kesteven	4	238
East Midlands	South Holland	5	237
East Midlands	South Derbyshire	6	234
East Midlands	Rutland	7	218
East Midlands	Rushcliffe	8	216
East Midlands	Oadby And Wigston	9	191
East Midlands	Nottingham	10	189
East Midlands	Northampton	11	186
East Midlands	North West Leicestershire	12	185
East Midlands	North Kesteven	13	179
East Midlands	North East Derbyshire	14	176
East Midlands	Newark and Sherwood	15	170
East Midlands	Melton	16	160
East Midlands	Mansfield	17	158
East Midlands	Lincoln	18	151
East Midlands	Leicester	19	147
East Midlands	Kettering	20	138
East Midlands	Hinckley And Bosworth	21	129

East Midlands	High Peak	22	127
East Midlands	Harborough	23	116
East Midlands	Gedling	24	105
East Midlands	Erewash	25	97
East Midlands	East Northamptonshire	26	87
East Midlands	East Lindsey	27	86
East Midlands	Derbyshire Dales	28	76
East Midlands	Derby	29	75
East Midlands	Daventry	30	74
East Midlands	Corby	31	63
East Midlands	Chesterfield	32	56
East Midlands	Charnwood	33	50
East Midlands	Broxtowe	34	39
East Midlands	Boston	35	25
East Midlands	Bolsover	36	23
East Midlands	Blaby	37	20
East Midlands	Bassetlaw	38	15
East Midlands	Ashfield	39	5
East Midlands	Amber Valley	40	3
East of England	Welwyn Hatfield	1	297
East of England	Waveney	2	293
East of England	Watford	3	292
East of England	Uttlesford	4	284
East of England	Thurrock	5	277
East of England	Three Rivers	6	276

East of England	Tendring	7	272
East of England	Suffolk Coastal	8	260
East of England	Stevenage	9	254
East of England	Southend-On-Sea	10	248
East of England	South Norfolk	11	240
East of England	South Cambridgeshire	12	233
East of England	Rochford	13	210
East of England	Peterborough	14	195
East of England	Norwich	15	188
East of England	North Norfolk	16	181
East of England	North Hertfordshire	17	178
East of England	Mid Suffolk	18	164
East of England	Maldon	19	155
East of England	Luton	20	153
East of England	King's Lynn and West Norfolk	21	139
East of England	Ipswich	22	134
East of England	Huntingdonshire	23	132
East of England	Hertsmere	24	126
East of England	Harlow	25	118
East of England	Great Yarmouth	26	109
East of England	Forest Heath	27	101
East of England	Fenland	28	100
East of England	Epping Forest	29	95
East of England	East Hertfordshire	30	85
East of England	East Cambridgeshire	31	81

East of England	Dacorum	32	71
East of England	Colchester	33	61
East of England	Chelmsford	34	51
East of England	Central Bedfordshire	35	49
East of England	Castle Point	36	48
East of England	Cambridge	37	43
East of England	Broxbourne	38	38
East of England	Broadland	39	35
East of England	Brentwood	40	32
East of England	Breckland	41	30
East of England	Braintree	42	29
East of England	Bedford	43	17
East of England	Basildon	44	13
East of England	Babergh	45	8
London	Westminster	1	305
London	Wandsworth	2	289
London	Waltham Forest	3	288
London	Tower Hamlets	4	281
London	Sutton	5	263
London	Southwark	6	249
London	Richmond Upon Thames	7	207
London	Redbridge	8	202
London	Newham	9	173
London	Merton	10	162
London	Lewisham	11	149

London	Lambeth	12	144
London	Kingston Upon Thames	13	141
London	Kensington and Chelsea	14	137
London	Islington	15	136
London	Hounslow	16	131
London	Hillingdon	17	128
London	Havering	18	125
London	Harrow	19	120
London	Haringey	20	117
London	Hammersmith and Fulham	21	115
London	Hackney	22	112
London	Greenwich	23	110
London	Enfield	24	94
London	Ealing	25	80
London	Croydon	26	70
London	Camden	27	44
London	Bromley	28	36
London	Brent	29	31
London	Bexley	30	18
London	Barnet	31	10
London	Barking and Dagenham	32	9
North East	Sunderland	1	261
North East	Stockton-On-Tees	2	256
North East	South Tyneside	3	246
North East	Redcar and Cleveland	4	203

North East	Northumberland	5	187
North East	North Tyneside	6	183
North East	Newcastle Upon Tyne	7	172
North East	Middlesbrough	8	166
North East	Hartlepool	9	122
North East	Gateshead	10	104
North East	Darlington	11	72
North East	County Durham	12	66
North West	Wyre	1	319
North West	Wirral	2	311
North West	Wigan	3	307
North West	West Lancashire	4	301
North West	Warrington	5	290
North West	Trafford	6	282
North West	Tameside	7	266
North West	Stockport	8	255
North West	St. Helens	9	251
North West	South Ribble	10	243
North West	South Lakeland	11	239
North West	Sefton	12	224
North West	Salford	13	220
North West	Rossendale	14	211
North West	Rochdale	15	209
North West	Ribble Valley	16	206
North West	Preston	17	199

North West	Pendle	18	194
North West	Oldham	19	192
North West	Manchester	20	157
North West	Liverpool	21	152
North West	Lancaster	22	145
North West	Knowsley	23	143
North West	Hyndburn	24	133
North West	Halton	25	113
North West	Fylde	26	103
North West	Eden	27	92
North West	Copeland	28	62
North West	Chorley	29	59
North West	Cheshire West and Chester	30	55
North West	Cheshire East	31	54
North West	Carlisle	32	47
North West	Bury	33	41
North West	Burnley	34	40
North West	Bolton	35	24
North West	Blackpool	36	22
North West	Blackburn With Darwen	37	21
North West	Barrow-In-Furness	38	12
North West	Allerdale	39	2
South East	Wycombe	1	318
South East	Worthing	2	316
South East	Wokingham	3	313

South East	Woking	4	312
South East	Windsor and Maidenhead	5	310
South East	Winchester	6	309
South East	West Oxfordshire	7	303
South East	West Berkshire	8	298
South East	Wealden	9	295
South East	Waverley	10	294
South East	Vale of White Horse	11	285
South East	Tunbridge Wells	12	283
South East	Tonbridge and Malling	13	278
South East	Thanet	14	275
South East	Test Valley	15	273
South East	Tandridge	16	268
South East	Swale	17	264
South East	Surrey Heath	18	262
South East	Spelthorne	19	250
South East	Southampton	20	247
South East	South Oxfordshire	21	242
South East	South Bucks	22	232
South East	Slough	23	230
South East	Shepway	24	228
South East	Sevenoaks	25	226
South East	Rushmoor	26	217
South East	Runnymede	27	215
South East	Rother	28	212

South East	Reigate and Banstead	29	205
South East	Reading	30	201
South East	Portsmouth	31	198
South East	Oxford	32	193
South East	New Forest	33	169
South East	Mole Valley	34	168
South East	Milton Keynes	35	167
South East	Mid Sussex	36	165
South East	Medway	37	159
South East	Maidstone	38	154
South East	Lewes	39	148
South East	Isle of Wight	40	135
South East	Horsham	41	130
South East	Havant	42	124
South East	Hastings	43	123
South East	Hart	44	121
South East	Guildford	45	111
South East	Gravesham	46	108
South East	Gosport	47	107
South East	Fareham	48	99
South East	Epsom and Ewell	49	96
South East	Elmbridge	50	93
South East	Eastleigh	51	91
South East	Eastbourne	52	90
South East	East Hampshire	53	84

South East	Dover	54	78
South East	Dartford	55	73
South East	Crawley	56	69
South East	Chiltern	57	58
South East	Chichester	58	57
South East	Cherwell	59	53
South East	Canterbury	60	46
South East	Brighton and Hove	61	33
South East	Bracknell Forest	62	27
South East	Basingstoke and Deane	63	14
South East	Aylesbury Vale	64	7
South East	Ashford	65	6
South East	Arun	66	4
South East	Adur	67	1
South West	Wiltshire	1	308
South West	Weymouth and Portland	2	306
South West	West Somerset	3	304
South West	West Dorset	4	300
South West	West Devon	5	299
South West	Torridge	6	280
South West	Torbay	7	279
South West	Tewkesbury	8	274
South West	Teignbridge	9	270
South West	Taunton Deane	10	269
South West	Swindon	11	265

South West	Stroud	12	259
South West	South Somerset	13	244
South West	South Hams	14	236
South West	South Gloucestershire	15	235
South West	Sedgemoor	16	223
South West	Purbeck	17	200
South West	Poole	18	197
South West	Plymouth	19	196
South West	North Somerset	20	182
South West	North Dorset	21	175
South West	North Devon	22	174
South West	Mid Devon	23	163
South West	Mendip	24	161
South West	Gloucester	25	106
South West	Forest of Dean	26	102
South West	Exeter	27	98
South West	East Dorset	28	83
South West	East Devon	29	82
South West	Cotswold	30	65
South West	Cornwall	31	64
South West	Christchurch	32	60
South West	Cheltenham	33	52
South West	Bristol, City of	34	34
South West	Bournemouth	35	26
South West	Bath and North East Somerset	36	16

West Midlands	Wyre Forest	1	320
West Midlands	Wychavon	2	317
West Midlands	Worcester	3	315
West Midlands	Wolverhampton	4	314
West Midlands	Warwick	5	291
West Midlands	Walsall	6	287
West Midlands	Telford and Wrekin	7	271
West Midlands	Tamworth	8	267
West Midlands	Stratford-On-Avon	9	258
West Midlands	Stoke-On-Trent	10	257
West Midlands	Staffordshire Moorlands	11	253
West Midlands	Stafford	12	252
West Midlands	South Staffordshire	13	245
West Midlands	Solihull	14	231
West Midlands	Shropshire	15	229
West Midlands	Sandwell	16	221
West Midlands	Rugby	17	214
West Midlands	Redditch	18	204
West Midlands	Nuneaton and Bedworth	19	190
West Midlands	North Warwickshire	20	184
West Midlands	Newcastle-Under-Lyme	21	171
West Midlands	Malvern Hills	22	156
West Midlands	Lichfield	23	150
West Midlands	East Staffordshire	24	89
West Midlands	Dudley	25	79

West Midlands	Coventry	26	67
West Midlands	Cannock Chase	27	45
West Midlands	Bromsgrove	28	37
West Midlands	Birmingham	29	19
Yorkshire and The Humber	York	1	321
Yorkshire and The Humber	Wakefield	2	286
Yorkshire and The Humber	Sheffield	3	227
Yorkshire and The Humber	Selby	4	225
Yorkshire and The Humber	Scarborough	5	222
Yorkshire and The Humber	Ryedale	6	219
Yorkshire and The Humber	Rotherham	7	213
Yorkshire and The Humber	Richmondshire	8	208
Yorkshire and The Humber	North Lincolnshire	9	180
Yorkshire and The Humber	North East Lincolnshire	10	177
Yorkshire and The Humber	Leeds	11	146
Yorkshire and The Humber	Kirklees	12	142
Yorkshire and The Humber	Kingston Upon Hull, City of	13	140
Yorkshire and The Humber	Harrogate	14	119
Yorkshire and The Humber	Hambleton	15	114
Yorkshire and The Humber	East Riding of Yorkshire	16	88
Yorkshire and The Humber	Doncaster	17	77
Yorkshire and The Humber	Craven	18	68
Yorkshire and The Humber	Calderdale	19	42
Yorkshire and The Humber	Bradford	20	28
Yorkshire and The Humber	Barnsley	21	11

Table A1: Local authority reference IDs look-up table.

Conditionally autoregressive (CAR) distribution

Suppose that $\omega_{jj'}$ is an element of a square $J \times J$ adjacency matrix, where $\omega_{jj'} = 1$ for j and j' sharing a boundary and $\omega_{jj'} = 0$ otherwise. Then we model the conditional distribution by

$$\varphi_j | \varphi_{j'} \sim N\left(\frac{\sum_{j' \neq j} \omega_{jj'} \varphi_{j'}}{\sum_{j' \neq j} \omega_{jj'}}, \frac{\sigma_\varphi^2}{\sum_{j' \neq j} \omega_{jj'}}\right)$$

Note that 2 of the LAs had no neighbours (Isle of Scilly and Isle of Wight).

Spatial variance partitioning

The variance partition coefficient (VPC) for the LA spatial components is

$$\lambda = \frac{\sigma_\varphi^2}{\sigma_\varphi^2 + \sigma_{la}^2}$$

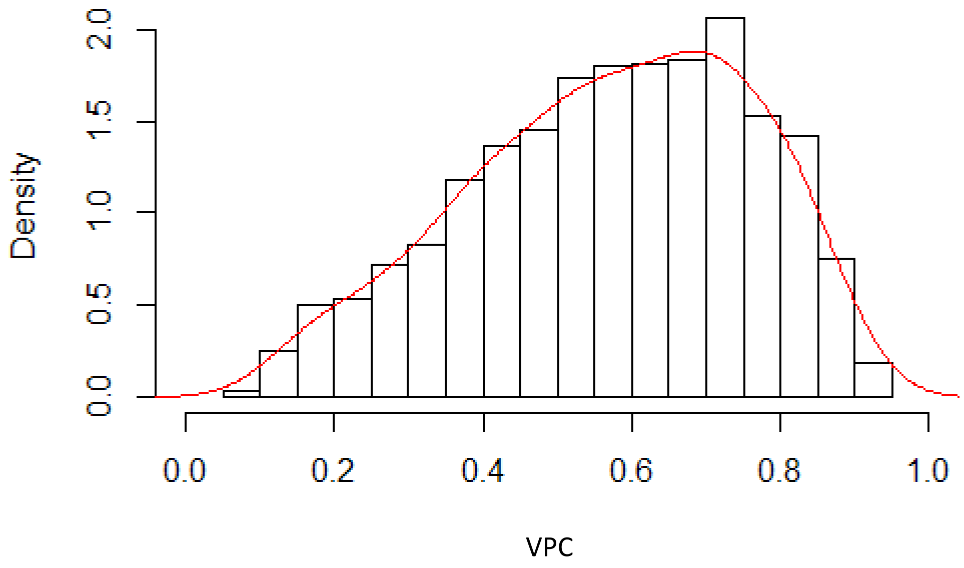

Figure A1: Posterior distribution of variance partition coefficient (VPC) for LA random effects.

From Figure A1, an LA's neighbouring LAs tends to (weakly) have a higher influence on the estimation of chlamydia testing in the previous year than the non-spatial structured LA level random effects.

Estimation of joint distribution

As discussed in the main text, the joint distribution of demographic and risk factors is estimated by combining conditional distributions obtained from available resources.

Due to limitations in the available data we make the following approximations,

$$p(age, sex, ethnic, student, livealone, LA) \approx p(age, sex, LA) \times p(ethnic|age, sex, LA) \times \frac{p(student|LA)}{E_{LA}[p(student|LA)]} \times p(student|age, sex) \times \frac{p(livealone|LA)}{E_{LA}[p(livealone|LA)]} \times p(livealone|age, sex)$$

Accounting for repeat testing within a year

Repeat testing statistics are taken from [1], which gives the number of test per person in a given year in the NCSP and GUMCAD data sets.

We required the total number of people that test in that year so needed to remove those people who were repeat testing. Without any loss of generality, let us assume that there are only one or two tests per person. The proportion of recorded tests which are first visits in a year is

$$prop(1\ test) + \frac{prop(2\ tests)}{2}$$

For males and females combined and NCSP data set [1],

Number of tests per person in 2010	Proportion
1	91%
2	8%

Thus, the adjustment is 91 + 8/2 = 95%. This proportion is used to scale the observed number of tests in the surveillance data, which is then compared with the MRP predictions.

Alternatively, it may be natural not to think in terms of scaling down the number of observed tests in the surveillance data but rather scaling up the population size to account for repeat testing to give an *effective* population size. In this case, we want the expected number of additional tests for an individual i.

$$E[number\ of\ new\ tests] = \sum i\ P(number\ of\ tests = i + 1)$$

Where, e.g.

$$P(number\ of\ tests = 2) = \frac{\frac{prop(2\ tests)}{2}}{prop(1\ test) + \frac{prop(2\ tests)}{2}}$$

Generally,

$$\sum \frac{prop(i\ tests)}{i} = \frac{1}{1 + E[number\ of\ new\ tests]}$$

We could further use subsets, e.g. GUMCAD and NCSP specific values, to calculate a weighted average depending on the relative amount of testing done in each testing venue for a given area e.g. LA. In practice, because the numbers are similar, this makes very little difference to the results.

Generalisations of the model

We also investigated the effect on model performance of separating-out those individuals who had not had sex in the previous year and had not tested for chlamydia and those that had had sex and not tested[2]. It may be that for particular covariate values, those individuals who had had sex would test. Of course, the reason for them having sex are also associated with the same group of covariates. Thus, we fit two logistic models, firstly with a response variable of whether they have had sex in the previous year and secondly whether they have tested for chlamydia in the previous year using only the sample of individuals who have had sex. The post-stratification equation is then

$$p_{la}^{tested} = \frac{\sum_{j \in S} N_j\, p_j^{hadsex} p_j^{tested}}{\sum_{j \in S} N_j}$$

A further generalisation is to include in the data model additional covariate values for which we do not have LA level data available with which to perform post-stratification. In this case, we can replace the missing values with the expected value from a second fit regressing the missing value variable on the remaining covariates. For example, if we wished to include the covariate 'if an individual had sex in the previous year' (*sex1yr*)

$$\Pr(y_i = 1) = logit^{-1}(\beta^0 + \alpha_{j[i]}^{age} + \alpha_i^{male} male_i + \alpha_{k[i]}^{ethnicity} + \alpha_i^{student} student_i$$

$$+\alpha_i^{livealone} livealone_i + \alpha_{m[i]}^{la} + \alpha_i^{sex1yr} sex1yr_i)$$

And

$$\Pr(sex1yr_i = 1)$$

$$= logit^{-1}\left(\beta^0 + \alpha_{j[i]}^{age} + \alpha_i^{male} male_i + \alpha_{k[i]}^{ethnicity} + \alpha_i^{student} student_i\right.$$

$$\left. + \alpha_i^{livealone} livealone_i + \alpha_{m[i]}^{la}\right)$$

Small numbers of observations

Generally, a binomial-logit model will suffice, as used in this analysis, but in applications where various marginal distributions in the data have few observations a quasi-Poisson distribution may be appropriate [3].

Model validation

Multi-collinearity was detected using the generalised variance inflation factor (GVIF) [4]. A GVIF of 2 - 5 indicates mild multi-collinearity. From Table A2 we see that there was little multi-collinearity in the model parameter.

Variable	GVIF	Degrees of Freedom
Sex	1.023	1
Age	1.359	1
Ethnic group	1.139	5
Live alone	1.043	1
Student	1.33	1
Conception decile	2.416	9
Urban-Rural classification	1.563	1
IMD upper quartile	2.092	1
Government region	1.309	1

Table A2: Generalised Variance Inflation Factor for each covariate used in the multilevel regression with post-stratification model.

Order of operations in MRP calculation

Depending on at which point we reduce the posterior distribution down to a point estimate may (slightly) alter the point estimate. That is, we may use the maximum a-posterior (MAP) estimates from the posterior distribution fitting to Natsal-3 data to calculate the final post-stratified LA level estimates

$$E_{LA}\left[\hat{\theta}_{MAP}\right]$$

Alternatively, we can propagate forward the whole posterior sample obtained from WinBUGS [5] and then take expectation at the very end

$$\widehat{E_{LA}[\theta_i^*]}$$

Exploratory data analysis plots

Figure A2 shows the scatterplots of LA level continuous covariates and a histogram for discrete valued covariates against 2011 NCSP coverage.

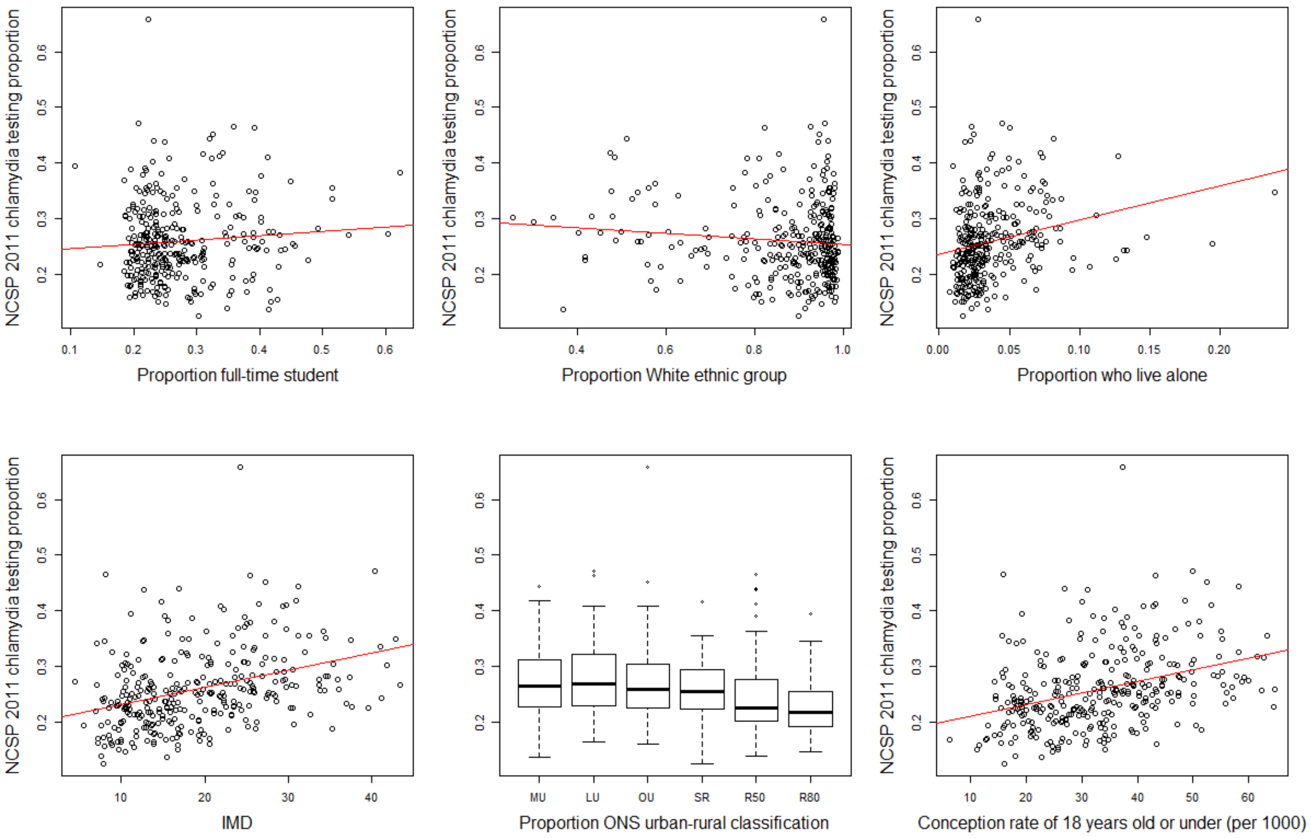

Figure A2: Scatterplots of LA level covariates and a boxplot for discrete values covariates against 2011 NCSP coverage. Linear regression fits are shown by red lines.

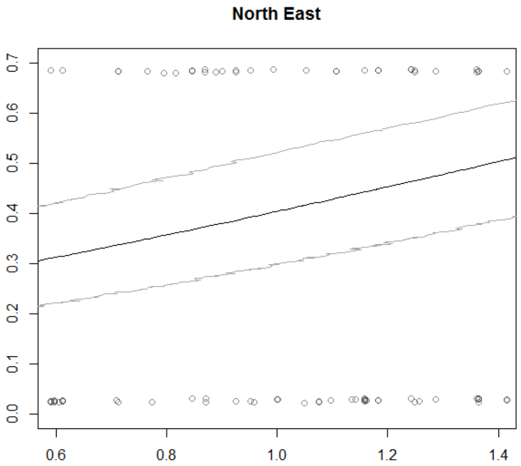
North East
0.0 0.1 0.2 0.3 0.4 0.5 0.6 0.7
0.6 0.8 1.0 1.2 1.4

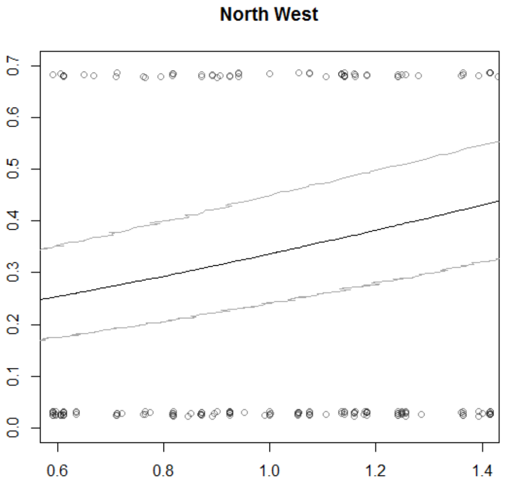
North West
0.0 0.1 0.2 0.3 0.4 0.5 0.6 0.7
0.6 0.8 1.0 1.2 1.4

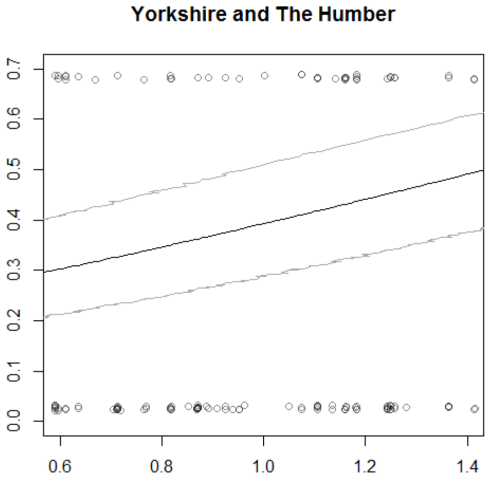
Yorkshire and The Humber
0.0 0.1 0.2 0.3 0.4 0.5 0.6 0.7
0.6 0.8 1.0 1.2 1.4

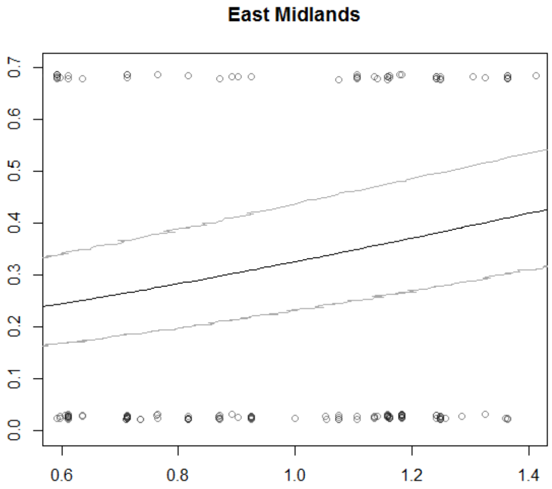
East Midlands
0.0 0.1 0.2 0.3 0.4 0.5 0.6 0.7
0.6 0.8 1.0 1.2 1.4

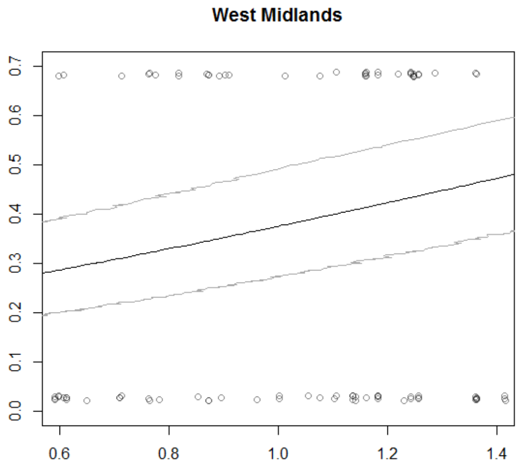
West Midlands
0.0 0.1 0.2 0.3 0.4 0.5 0.6 0.7
0.6 0.8 1.0 1.2 1.4

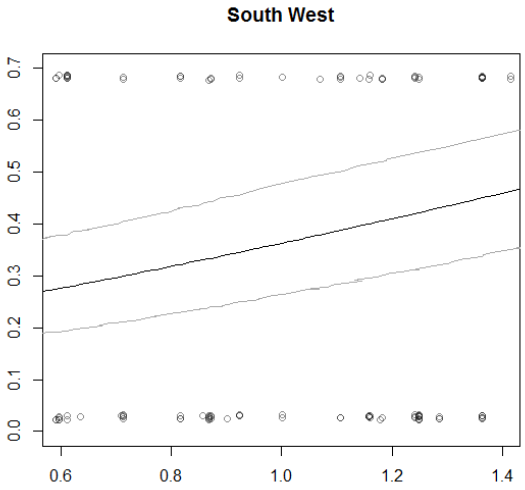
South West
0.0 0.1 0.2 0.3 0.4 0.5 0.6 0.7
0.6 0.8 1.0 1.2 1.4

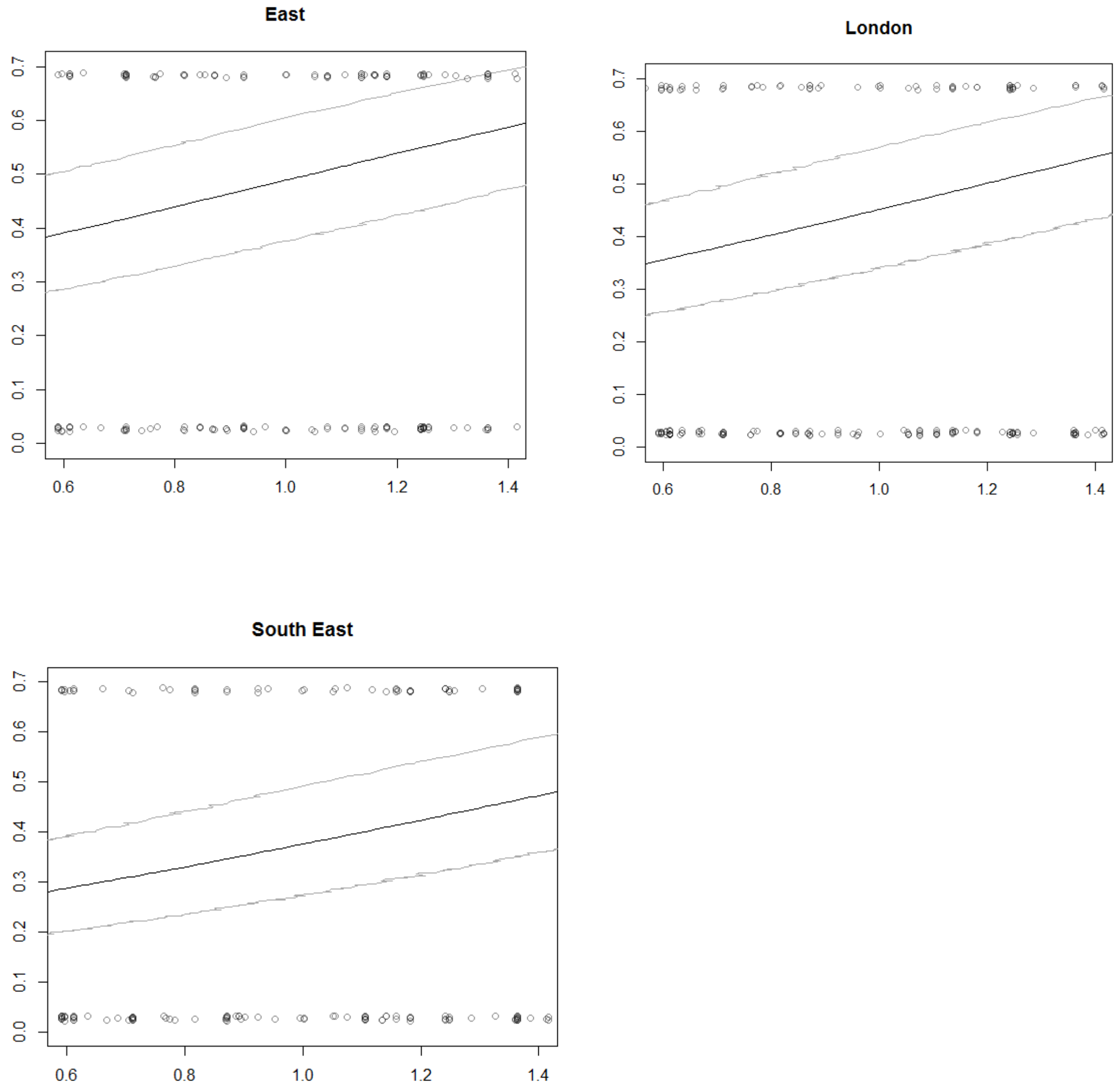

Figure A3: Logistic model predictions for the probability of testing for chlamydia stratified by government region. The bold central line is the mean predicted probability and the lighter lines are the 2 standard error upper and lower bounds [6]. Points at probability 0 and 1 are the direct Natsal-3 values for whether an individual was tested for chlamydia in the previous year. The x-axis values are the logistic model fits for the model without government region.

- The probability of testing for chlamydia in the last year ranges from about 30% to 50% within government regions.
- East and London have a relatively higher upper value of over 50%.

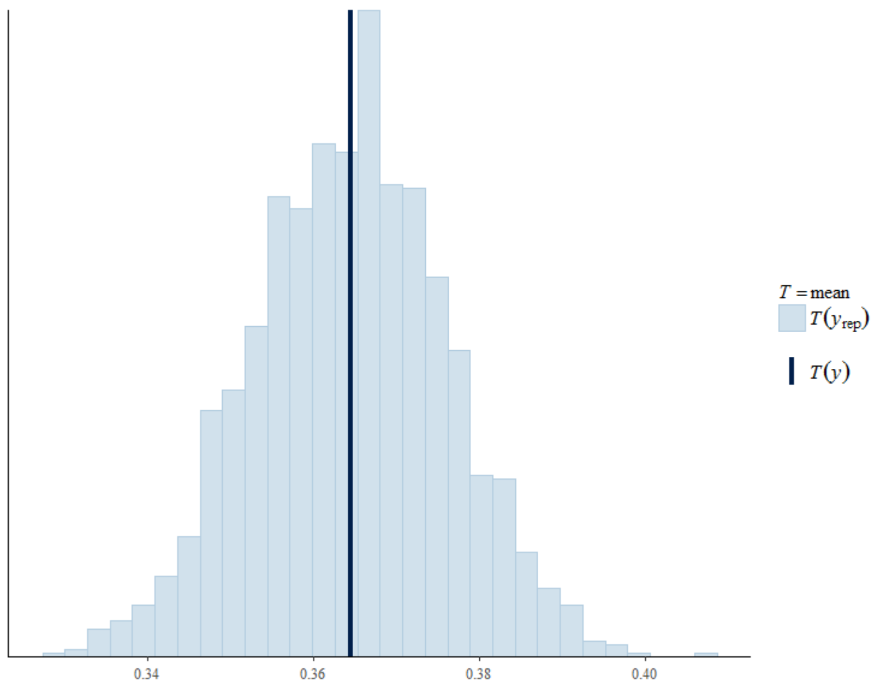

Figure A4: Distribution of the pooled proportion of individuals who tested for chlamydia over the replicated datasets from the posterior predictive distribution and compared to the observed total proportion in Natsal-3.

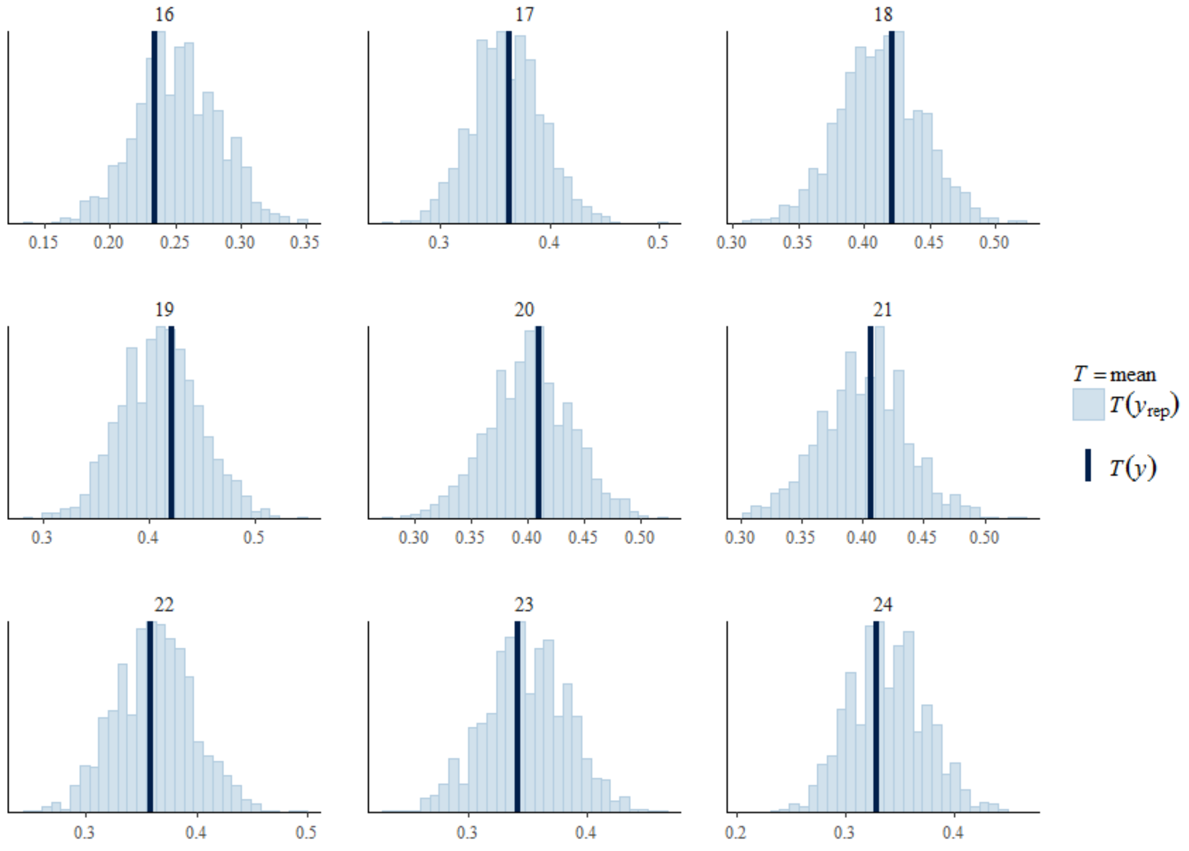

Figure A5: Distribution of the proportion of individuals who tested for chlamydia by age group over the replicated datasets from the posterior predictive distribution and compared to the observed proportion in Natsal-3.

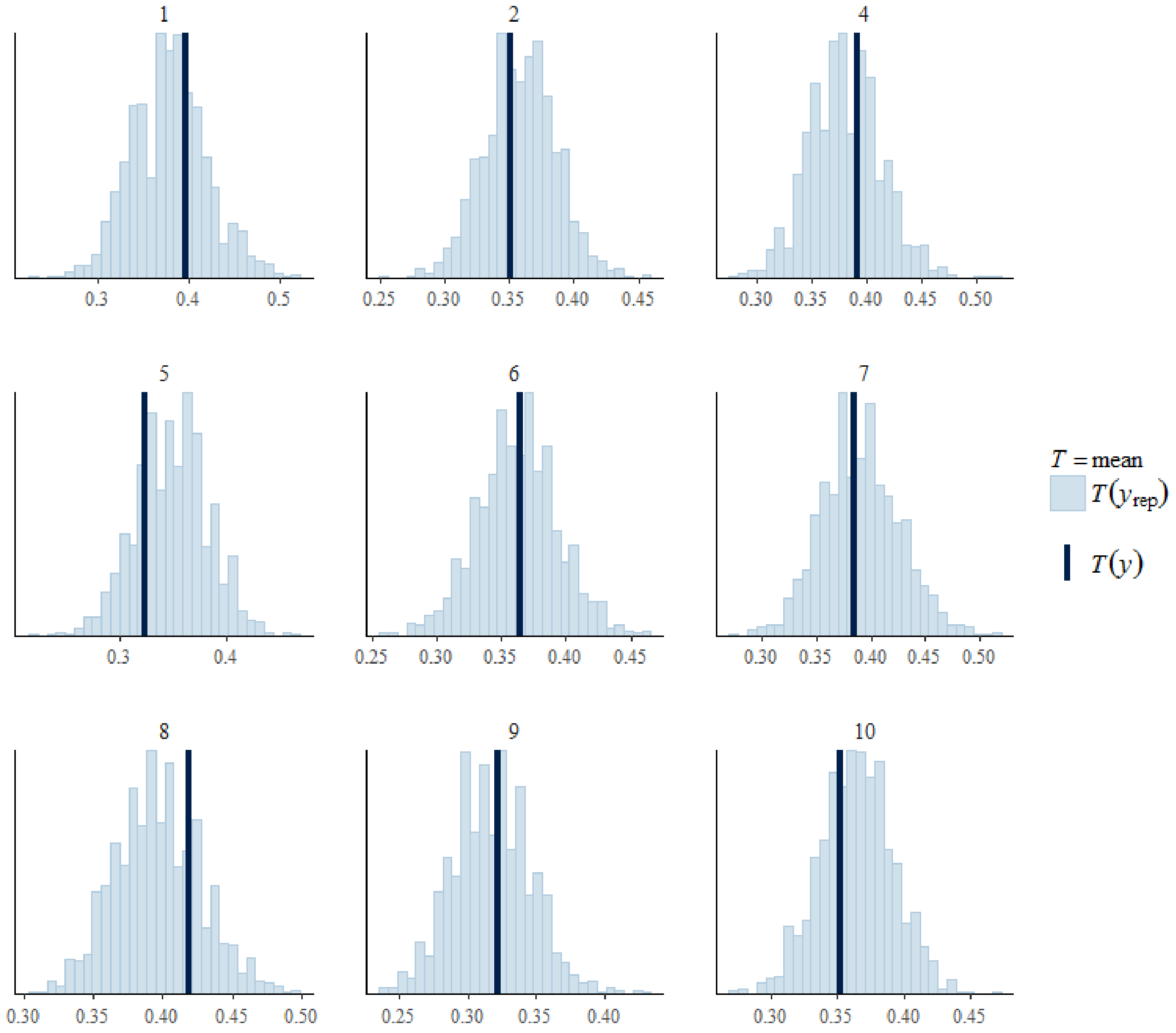

Figure A6: Distribution of the proportion of individuals who tested for chlamydia by region over the replicated datasets from the posterior predictive distribution and compared to the observed proportion in Natsal-3.

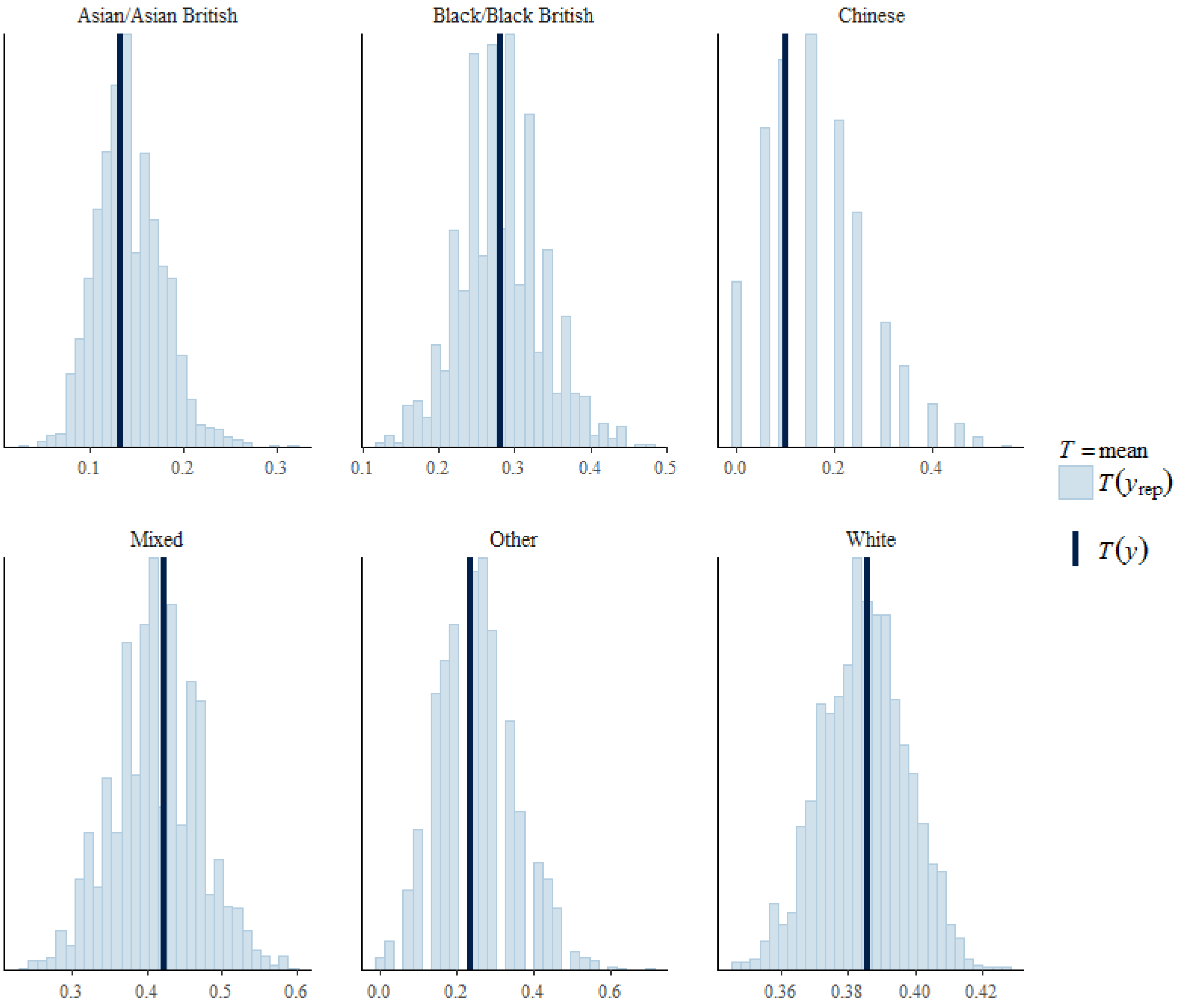

Figure A7: Distribution of the proportion of individuals who tested for chlamydia by ethnic group over the replicated datasets from the posterior predictive distribution and compared to the observed proportion in Natsal-3.

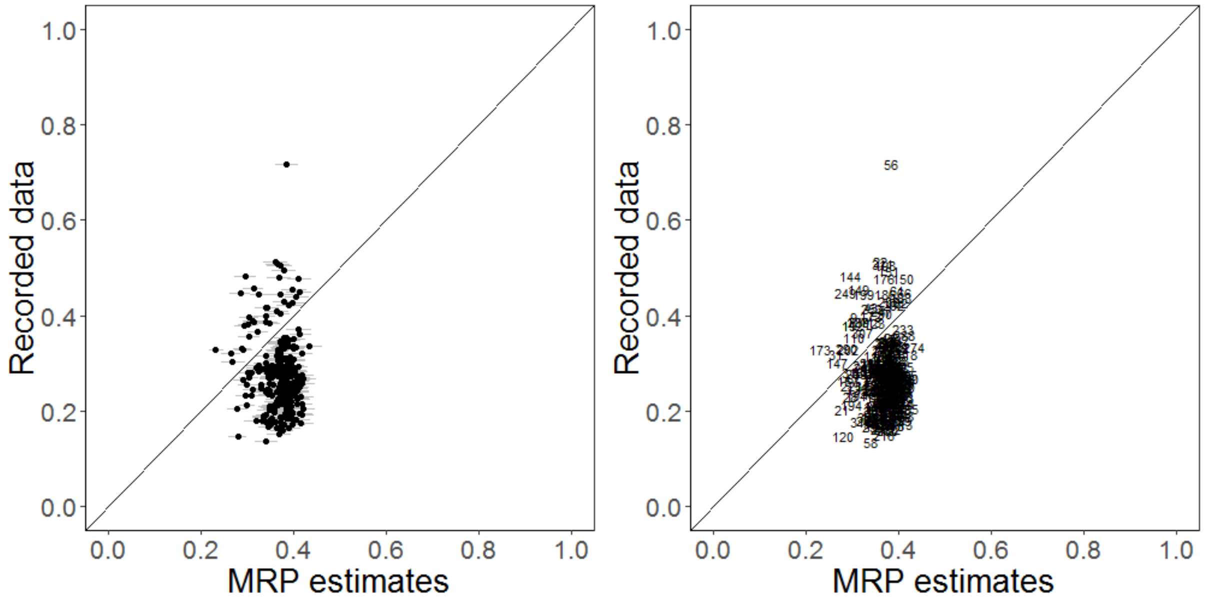

Figure A8: Posterior predictions of chlamydia testing coverage in the previous year, showing the 50% Credible Intervals (grey lines) for each LA propagating forward the uncertainty associated with each parameter estimate. Black points are the maximum a-posteriori estimates. The number represent different LAs (see Table A1 for names).

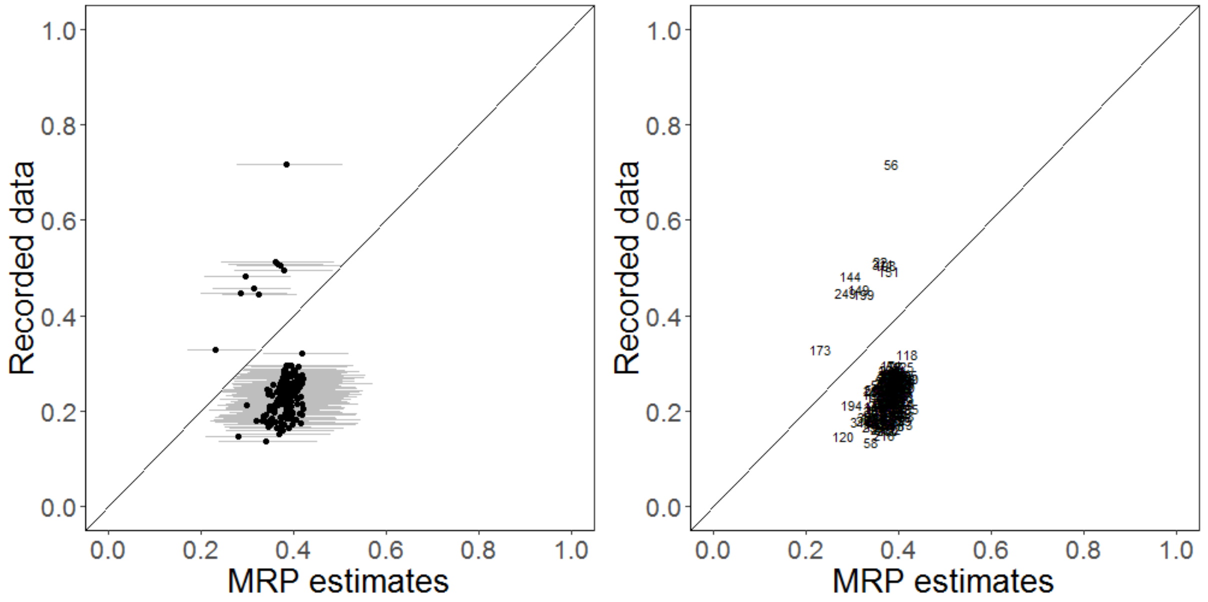

Figure A9: Posterior predictions of chlamydia testing coverage in the previous year, showing the 99% Credible Intervals (grey lines) for each LA propagating forward the uncertainty associated with each parameter estimate. Black points are the maximum a-posteriori estimates. The number represent different LAs (see Table A1 for names).

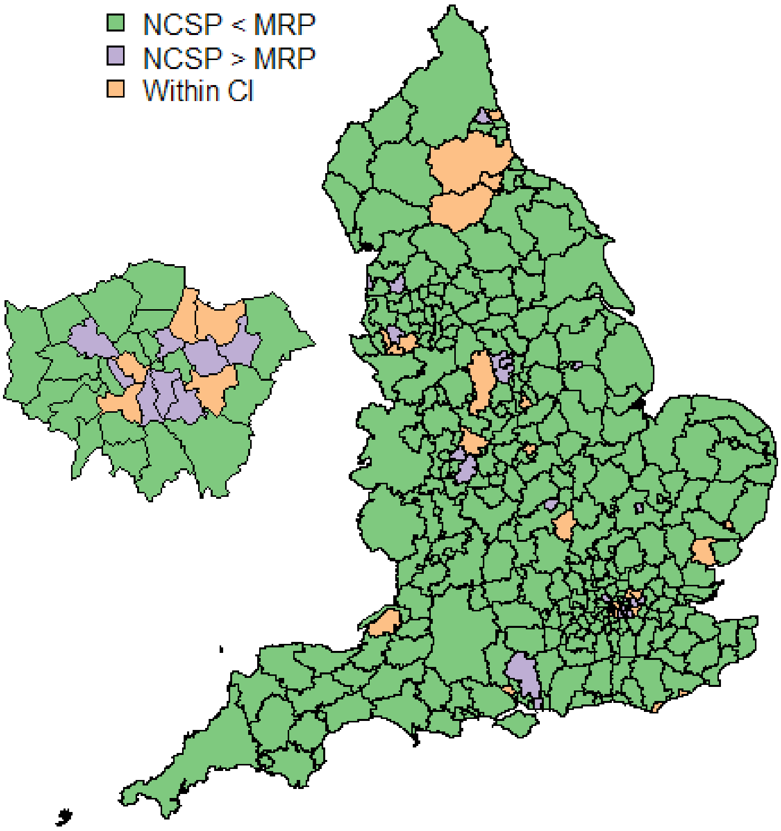

Figure A10: A choropleth map of England by LA. LAs within (orange) and outside of the posterior distribution 50% credible interval. Higher MRP estimates to NCSP 2011 are in green and lower MRP estimates to NCSP 2011 are in purple. The majority of the time the MRP over-estimates testing relative to recorded testing.

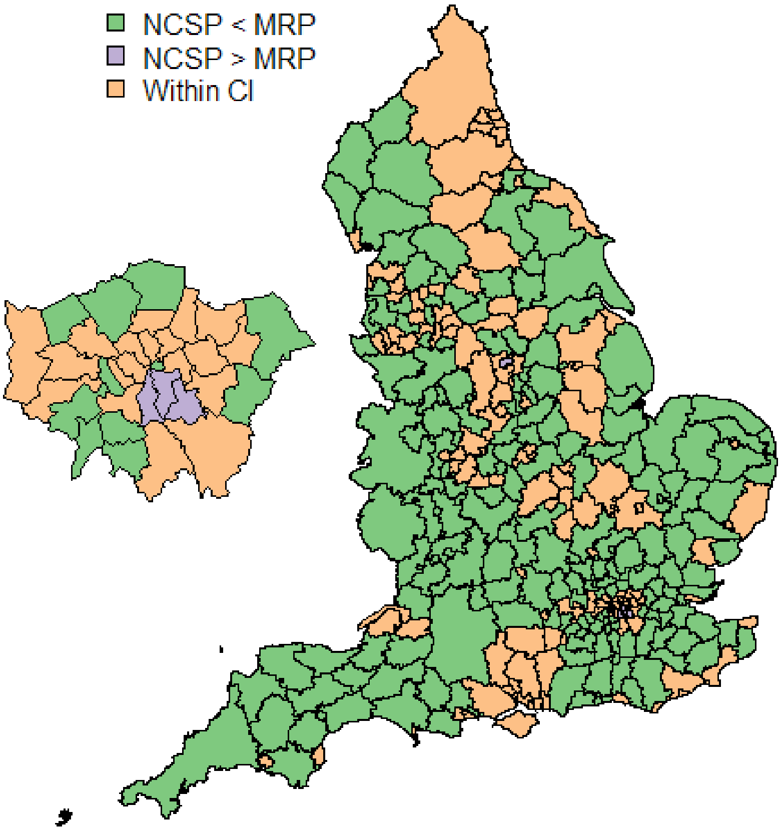

Figure A11: A choropleth map of England by LA. LAs within (orange) and outside of the posterior distribution 99% credible interval. Higher MRP estimates to NCSP 2011 are in green and lower MRP estimates to NCSP 2011 are in purple. MRP model estimates generally over-estimated relative to registered testing for more rural regions of England but within the 99% credible intervals in more urban places like London and Manchester, for example.

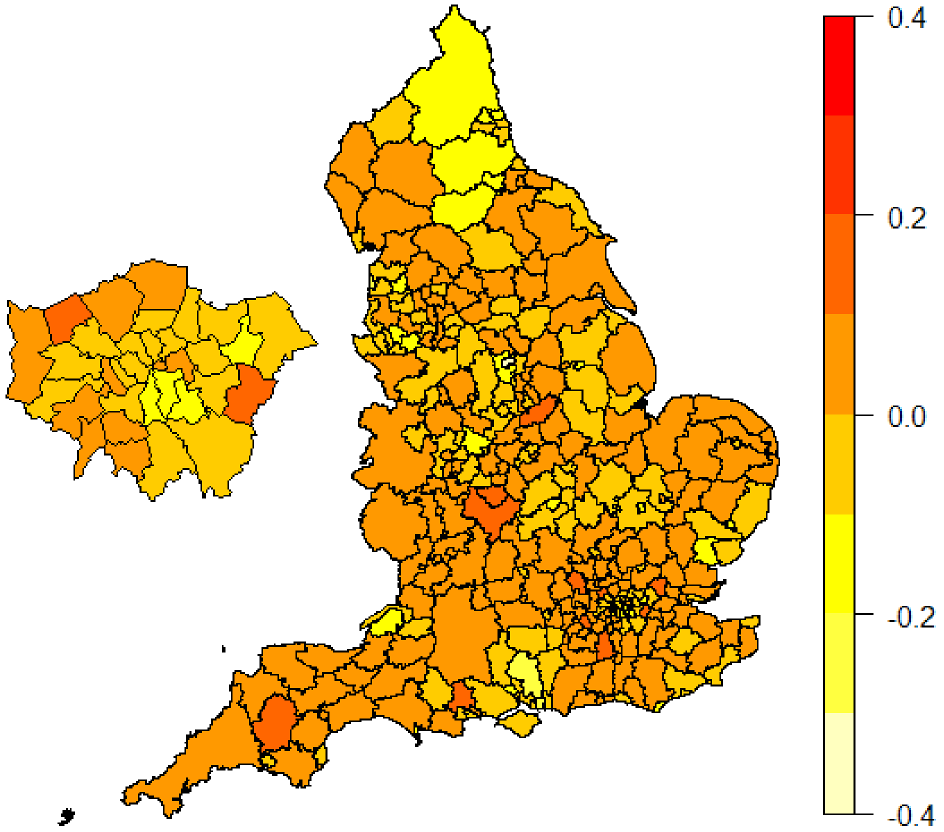

Figure A12: A choropleth map of England by LA of the difference between the national mean and each LAs chlamydia testing in the previous year (coverage) from the recorded testing NCSP 2011.

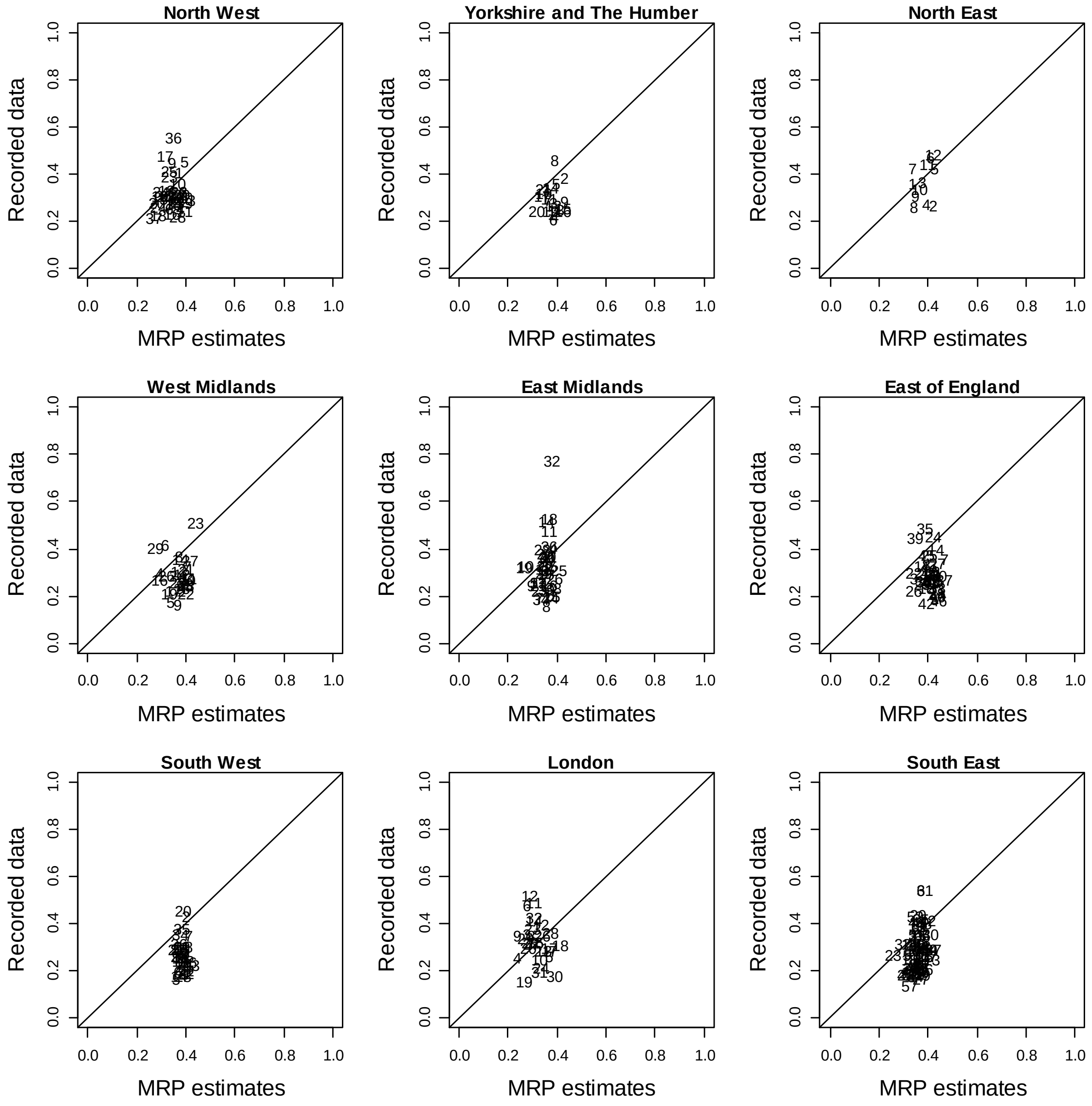

Figure A13: Estimated chlamydia testing coverage in the previous year for MRP against NCSP 2011 surveillance data, stratified by government region. The diagonal lines represent equality. The numbers correspond to different LAs (see Table A1 for names).

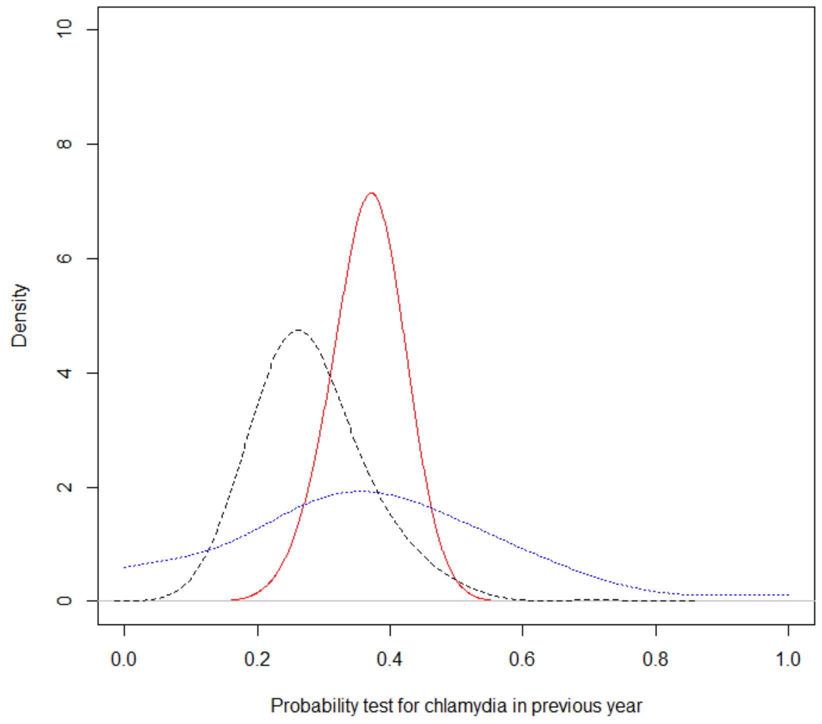

Figure A14: Distributions of probability testing for chlamydia in previous year by LA for i) MRP estimation (solid red line); ii) Natsal-3 direct estimate (dotted blue line) and (iii) NCSP 2011 (dashed black line).

**Model formula***	**Df**	**AIC**	**BIC**	**Log-likelihood**	**Deviance**	χ^2	χ^2 **Df**	**Pr(>χ^2)**
cttestly ~ 1 + (1 | age)	2	7814.38	7829.04	-3905.19	7810.38	-	-	-
cttestly ~ 1 + (1 | gor)	2	9794.58	9809.24	-4895.29	9790.58	0.00	0	1
cttestly ~ 1 + sex	3	9139.75	9161.73	-4566.88	9133.75	656.83	1	7.31E-145
cttestly ~ (1 | age) + sex	3	4392.46	4414.44	-2193.23	4386.46	4747.29	0	0
cttestly ~ (1 | age) + sex + ethnic	4	4389.91	4419.22	-2190.95	4381.91	4.55	1	0.0329
cttestly ~ (1 | age) + sex + student	4	4390.50	4419.82	-2191.25	4382.50	0.00	0	1
cttestly ~ (1 | age) + sex + Livealone	4	4380.01	4409.32	-2186.01	4372.01	10.49	0	0
cttestly ~ (1 | age) + sex + ethnic:student	5	4389.96	4426.60	-2189.98	4379.96	0.00	1	1
cttestly ~ sex + (sex | age)	5	4394.77	4431.41	-2192.39	4384.77	0.00	0	1
cttestly ~ sex + (student | age)	5	4389.86	4426.50	-2189.93	4379.86	4.91	0	0
cttestly ~ sex + (ethnic | age)	5	4389.39	4426.03	-2189.70	4379.39	0.46	0	0
cttestly ~ sex + (Livealone | age)	5	4390.29	4426.93	-2190.14	4380.29	0.00	0	1
cttestly ~ (1 | age) + sex + ethnic + student + Livealone	6	4376.27	4420.23	-2182.13	4364.27	16.02	1	6.26E-05
cttestly ~ (1 | age) + sex + ethnic + student + Livealone + (1 | gor)	7	4378.27	4429.56	-2182.13	4364.27	0.00	1	1

cttestly ~ (1 | age) + sex + ethnic + student + Livealone + (1 | gor) + (1 | Conception.decile)	8	4379.90	4438.53	-2181.95	4363.90	0.36	1	0.547847808
cttestly ~ (1 | age) + sex + ethnic + student + Livealone + (1 | gor) + (1 | `Numerical classification`)	8	4380.27	4438.89	-2182.13	4364.27	0.00	0	1
cttestly ~ (1 | age) + sex + ethnic + student + Livealone + (1 | gor) + UpperQ_IMD	8	4380.10	4438.73	-2182.05	4364.10	0.16	0	0
cttestly ~ (1 | age) + sex + ethnic + student + Livealone + (1 | gor) + (1 | metcounty_UA)	8	4379.64	4438.27	-2181.82	4363.64	0.46	0	0
cttestly ~ (1 | age) + sex + ethnic + student + Livealone + (1 | gor) + (1 | Conception.decile) + (1 | `Numerical classification`) + UpperQ_IMD + (1 | metcounty_UA)	11	4385.38	4465.98	-2181.69	4363.38	0.27	3	0.965860357

*Table A3: Multi-level logistic regression model selection statistics using combined Natsal-3, administrative and ONS data.*R notation.*

‘Livealone’ variable is included to account for Natsal-3’s complex survey design which included household size. Very little research has been carried-out focusing on the relationship between household size and sexual health or sexual behaviour. An association has been shown between not living with family and having had an STI in girls[7].

References

1. Woodhall, S. C. *et al.* Repeat genital Chlamydia trachomatis testing rates in young adults in England, 2010. *Sex. Transm. Infect.* 51–56 (2013).
2. Park, D. K., Gelman, A. & Bafumi, J. Bayesian Multilevel Estimation with Poststratification : State-Level Estimates from National Polls. *Polit. Anal.* **12,** 375–385 (2004).
3. Ghitza, Y. & Gelman, A. Deep Interactions with MRP: Election Turnout and Voting Patterns Among Small Electoral Subgroups. *Am. J. Pol. Sci.* **57,** 762–776 (2013).
4. Fox, J. & Monette, G. Generalized Collinearity Diagnostics. *J. Am. Stat. Assoc.* **87,** 178–183 (1992).
5. Lunn, D. J., Thomas, A., Best, N. & Spiegelhalter, D. WinBUGS – a Bayesian modelling framework: concepts, structure, and extensibility. *Stat. Comput.* **10,** 325–337 (2000).
6. Park, D. K., Gelman, A. & Bafumi, J. State-level opinions from national surveys: Poststratification using multilevel logistic regression. *Public Opin. State Polit.* 209–228 (2006).
7. Kim, S. & Lee, C. Factors Affecting Sexually Transmitted Infections in South Korean High School Students. *Public Health Nurs.* **33,** 179–188 (2016).
